# Gut microbiota in chronic kidney disease-mineral and bone disorder: shared mechanisms, disease-specific signatures, and therapeutic prospects

**DOI:** 10.3389/fendo.2026.1802845

**Published:** 2026-04-13

**Authors:** Qianwei Wang, Zhicheng Zhou, Liang Pang, Yuchen Du, Xiaolong Li, Lijuan Dai

**Affiliations:** 1 Graduate School, Heilongjiang University of Chinese Medicine, Harbin, Heilongjiang, China; 2 Anorectal Department, The First Affiliated Hospital of Anhui University of Chinese Medicine, Hefei, Anhui, China; 3 Orthopedics Department, Wenzhou Hospital of Traditional Chinese Medicine Affiliated to Zhejiang University of Chinese Medicine, Wenzhou, Zhejiang, China; 4 Orthopedics Department, The First Affiliated Hospital of Anhui University of Chinese Medicine, Hefei, Anhui, China; 5 Orthopedics Department, Nanjing University of Chinese Medicine, Nanjing, Jiangsu, China; 6 Nephrology Department, The First Affiliated Hospital of Heilongjiang University of Chinese Medicine, Harbin, Heilongjiang, China

**Keywords:** chronic kidney disease-mineral and bone disorder (CKD-MBD), FGF23-klotho axis, gut microbiota, gut-kidney-bone axis, parathyroid hormone (PTH)

## Abstract

Chronic Kidney Disease-Mineral and Bone Disorder (CKD-MBD) is a systemic syndrome characterized by mineral metabolism disorders and impaired bone homeostasis. Recent studies have indicated that gut microbiota dysbiosis is a key regulatory factor driving the development and progression of this disease. This review systematically summarizes the mechanisms by which gut microbiota acts in CKD-MBD through the “gut-kidney-bone axis”: dysbiosis drives chronic low-grade inflammation by impairing the intestinal barrier and promoting endotoxin translocation; alterations in its metabolites (e.g., reduced short-chain fatty acids, accumulation of uremic toxins) and dysregulation of endocrine pathways (e.g., FGF23-Klotho axis, PTH) collectively exacerbate renal injury and abnormal bone metabolism. Additionally, in diseases such as CKD, rheumatoid arthritis (RA), osteoarthritis (OA), and osteoporosis (OP), gut microbiota exhibits the coexistence of “shared dysbiosis” and “disease-specific characteristics,” which collectively contribute to chronic inflammation and metabolic disorders. Interventional strategies targeting gut microbiota have demonstrated the potential to regulate this axis and improve bone health, marking that the management of metabolic bone diseases and chronic kidney disease is entering the “era of microbiome medicine.” This review aims to provide new insights into understanding the comorbidity mechanisms of the aforementioned diseases and lay a theoretical foundation for the development of microbiota-targeted therapeutic strategies.

## 1 Introduction

In the sophisticated “superorganism” that is the human body, the gut microbiota has evolved from once obscure symbiotic inhabitants into a core “active organ” regulating host health and disease. Over 100 trillion microbial cells, with their vast genetic reservoir and metabolic potential, deeply engage in nutrient metabolism, immune modulation, and barrier defense, forming the cornerstone of maintaining internal homeostasis (1). However, when this complex microecosystem becomes dysregulated, its impacts extend far beyond the gastrointestinal tract. Mounting evidence indicates that structural and functional abnormalities of the gut microbiota are key drivers of the development and progression of various chronic diseases, which has propelled research on the mechanisms linking the gut to distant organs into a new phase (2). Against this backdrop, chronic kidney disease (CKD) and metabolic bone diseases—two disease categories with globally rising prevalence that impose a heavy burden on public health systems—have garnered unprecedented attention regarding their associations with the gut microbiota. Traditionally, the progressive renal function decline in CKD and the osteoarticular damage in osteoarthritis (OA), osteoporosis (OP), and rheumatoid arthritis (RA) were classified into distinct pathological categories. However, recent studies have revealed that they share the core pathological backdrop of “chronic low-grade inflammation,” and gut microbiota dysbiosis is one of the key contributors to this backdrop (3). In CKD, gut microbiota dysbiosis drives the accumulation of uremic toxins and exacerbates renal injury; in OA, OP, and RA, the microbiota directly contributes to the imbalance between bone resorption and formation by regulating immunity and influencing metabolism (4–6).

More profoundly, the gut, kidneys, and bones are not independent battlefields but are closely interconnected through an intricate network termed the “gut-kidney-bone axis.” As the core hub, the gut microbiota enables bidirectional crosstalk among these three organs via its metabolites, immunomodulatory signals, and regulation of endocrine pathways. Dysregulation of this axis is regarded as a core mechanism underlying complex syndromes such as chronic kidney disease-mineral and bone disorder (CKD-MBD). This review aims to systematically synthesize the latest advances in this cutting-edge field. We will provide an overview of the core functions of the gut microbiota and the general hazards of dysbiosis; delve into the disease-specific dysbiosis characteristics and mechanisms of the gut microbiota in CKD and skeletal diseases including RA, OA, and OP, respectively; and focus on the integrative concept of the “gut-kidney-bone axis” to elaborate on how the gut microbiota acts as a bridge to mediate the crosstalk between the kidneys and bones, thereby offering novel perspectives for understanding the comorbidity mechanisms of these diseases.

## 2 Methods

To establish the evidence base for this review, we systematically searched three core databases: PubMed, Web of Science, and Embase, supplemented by manual searches on Google Scholar to retrieve gray literature. The search strategy combined Medical Subject Headings (MeSH terms) and free-text terms, focusing on “chronic kidney disease (CKD)”, “gut microbiota/microbiome”, “mineral and bone disorder (MBD)”, “gut-kidney-bone axis”, “rheumatoid arthritis (RA)”, “osteoarthritis (OA)”, “osteoporosis (OP)”, “short-chain fatty acids (SCFAs)”, “uremic toxins” (e.g., indoxyl sulfate, p-cresyl sulfate, trimethylamine N-oxide [TMAO]), and “gut-targeted interventions” (e.g., probiotics, prebiotics, synbiotics, dietary fiber, fecal microbiota transplantation [FMT]). This strategy aimed to comprehensively cover the multidimensional associations between gut microbiota remodeling and CKD-MBD as well as its related osteoarticular diseases.

## 3 Gut microbiota

There are at least 35,000 distinct species of microorganisms in the human body, among which the gastrointestinal tract harbors a highly complex and dynamic microbial community consisting of over 100 trillion microbial cells, predominantly composed of the phyla Firmicutes and Bacteroidetes (7). As an important symbiotic partner of the human body, the gut microbiota plays a pivotal role in maintaining host health, including participating in nutrient metabolism, regulating immune system function, and resisting pathogen invasion (1).

At the level of nutrient metabolism, the gut microbiota produces short-chain fatty acids (SCFAs) such as acetate, propionate, and butyrate by digesting dietary components not absorbed by the host. These metabolites not only serve as an energy source for the host but also help enhance intestinal barrier integrity, regulate systemic immune responses, and suppress inflammation (2, 8). The gut microbiota is also involved in the transformation and recycling of bile acids, as well as the synthesis of various nutrients including folic acid, vitamin K, and vitamin B12 (9). Current studies have found that *Prevotella* and *Ruminococcus* are involved in the synthesis of folic acid, vitamin K, and vitamin B12; *Bifidobacterium* is the primary producer of folic acid (10); *Lactobacillus* is an important strain for vitamin B12 production; and *Faecalibacterium prausnitzii* and *Akkermansia muciniphila* can produce anti-inflammatory metabolites (e.g., indole derivatives and conjugated linoleic acid) to enhance mucosal immunity (2). In addition, commensal bacteria maintain ecological balance through competitive exclusion of pathogens and secretion of antimicrobial peptides (e.g., bacteriocins) (11).

From the perspective of immune regulation, the gut microbiota plays a central role in regulating local and systemic immune responses through close interaction with the host’s immune system. Pattern recognition receptors (PRRs), such as Toll-like receptors (TLRs) and Nod-like receptors (NLRs), can recognize microbe-associated molecular patterns (MAMPs) and thereby activate downstream immune signaling pathways (12). As a key class of transmembrane pattern recognition receptors, TLRs not only play a critical role in microbial recognition and innate immune regulation but also help maintain intestinal epithelial homeostasis and resist damage. These receptors can specifically recognize molecular effectors produced by the gut microbiota, improve inflammatory bowel disease through related mechanisms, distinguish commensal bacteria from pathogenic bacteria, and regulate the expression levels of immune cells or their PRRs (13). Furthermore, a healthy gut microbiota contributes to enhancing epithelial barrier function; studies using mouse models have found that the colonic mucus layer of germ-free mice is thinner, while exposure to bacterial products rapidly restores mucus thickness to the level of conventionally raised mice, indicating that microbial signals play an important role in the establishment and maintenance of the mucus barrier (14).

Meanwhile, the gut microbiota also plays a central role in immune system development, especially the exposure to microorganisms in early life stages to shape immune tolerance and responsiveness. Specific bacteria such as *Bacteroides* and *Lactobacillus* can promote the differentiation of regulatory T cells (Tregs), thereby suppressing inflammation and preventing autoimmune diseases (13, 15, 16). The microbiota also interacts with gut-associated lymphoid tissue (GALT) to promote the secretion of immunoglobulin A (IgA), further enhancing mucosal immunity and defense against pathogens (17).

### 3.1 Dysbiosis

Combined with the latest research insights from the 2026 consensus statement on gut health by the International Scientific Association for Probiotics and Prebiotics (ISAPP), intestinal dysbiosis is not simply an abnormal change in the abundance of intestinal microbiota components. Rather, it is a comprehensive homeostatic disruption of the gut microbial ecosystem at the levels of compositional structure, metabolic function, and microbial–host interaction networks, representing a pathological state synergistically driven by microbiome dysfunction and aberrant host physiological responses. This updated perspective discards the traditional dichotomous view of “reduced beneficial bacteria and increased pathogens”, and instead emphasizes core abnormalities in ecological function and microbe–host crosstalk. Traditional predisposing factors remain diets high in sugar and fat, overuse of broad-spectrum antibiotics, and insufficient dietary fiber intake (3). Moreover, the latest consensus further clarifies that diverse endogenous and exogenous factors—including aging, early-life exposure, psychological stress, physical inactivity, and chronic diseases such as chronic kidney disease (CKD)—can collectively disrupt the homeostatic balance of the microbial ecosystem and trigger dysbiosis (18). The most common manifestations of dysbiosis are decreased levels of beneficial bacteria (e.g., *Faecalibacterium* and *Akkermansia*) and excessive growth of potential pro-inflammatory taxa/pathogens (e.g., Enterobacteriaceae and *Fusobacterium nucleatum*) (19, 20).

Dysbiosis is often accompanied by reduced beneficial metabolites and production of pathogenic metabolites (e.g., indoxyl sulfate [IS], p-cresyl sulfate [pCS], trimethylamine N-oxide [TMAO], and lipopolysaccharide [LPS]) (21). For instance, among these gut-derived uremic toxins, indoxyl sulfate (IS) represents the most prototypical indole-containing uremic toxin and plays a pivotal role in chronic kidney disease (CKD). IS is generated by gut bacteria through the metabolism of dietary tryptophan, undergoes sulfation modification in the liver, and is then excreted by the kidneys. In the state of CKD, impaired renal clearance leads to massive accumulation of IS in the circulation (22). As a key ligand of the aryl hydrocarbon receptor (AhR), IS can activate the AhR–NF-κB and MAPK signaling pathways, inducing oxidative stress, inflammatory cascades, and renal fibrosis (23). IS mediates endothelial dysfunction, vascular calcification, and myocardial fibrosis, and promotes the development of multiple cardiovascular disease phenotypes in CKD patients (24–26). IS also contributes to the pathogenesis of CKD-mineral and bone disorder (CKD-MBD) and renal anemia by impairing bone metabolism and erythropoiesis (27, 28). TMAO, a metabolite of dietary choline, has been shown in recent studies to promote macrophage foam cell formation and platelet aggregation, thereby exacerbating atherosclerosis (29). pCS impairs the integrity of the blood-brain barrier through the epidermal growth factor receptor (EGFR)/signal transducer and activator of transcription 3 (STAT3) signaling pathway (30). Microbial products such as LPS and flagellin translocate into the circulatory system, activating Toll-like receptors (TLRs) and Nod-like receptors (NLRs), which triggers activation of the NF-κB signaling pathway and systemic low-grade inflammation, further damaging intestinal barrier function (12).

Antibiotic exposure is a key factor contributing to dysbiosis. Scientists have found that antibiotic use in early life (during pregnancy and the neonatal period) significantly alters the microbiota composition—increasing the abundance of Proteobacteria and Firmicutes, decreasing Bacteroidetes and Actinobacteria (31, 32) —and elevates the risk of future allergies, obesity, neurological diseases, and cancer (33).

In critically ill patients, intestinal microecological imbalance is more pronounced, characterized by decreased Firmicutes and Bacteroidetes, increased Proteobacteria, reduced beneficial bacteria (anaerobes and *Lactobacillus*), and expanded pathogenic bacteria (*Enterococcus* and *Pseudomonas*) (34, 35).

In summary, the gut microbiota is no longer a passively accompanying “symbiont” in the traditional sense, but an active regulator that plays a key driving role in the occurrence and progression of various diseases.

## 4 Stage-specific and functional dysbiosis characteristics of gut microbiota in chronic kidney disease

Chronic kidney disease (CKD) is a chronic progressive disease characterized by progressive renal function decline and renal structural damage. With a persistently high global incidence, it is often complicated by severe complications such as cardiovascular diseases and cognitive impairments, imposing a heavy burden on patients’ physical and mental health as well as the global healthcare system (36, 37). Gut microbiota dysbiosis has been confirmed by numerous clinical and basic studies as a key participant in the pathophysiological process of CKD. It not only plays a critical regulatory role in disease initiation, progression acceleration, and complication development but also serves as a potential early diagnostic microbial biomarker and personalized therapeutic target.

### 4.1 Gut microbiota composition characteristics in CKD patients: stage-specific and universal patterns

Multicenter cohort studies and multi-omics analyses have verified that the gut microbiota of CKD patients exhibits significant structural and functional dysbiosis at the phylum, genus, and species levels. This dysbiosis shows clear stage-specificity as the disease progresses from early stages to end-stage renal disease (ESRD), while also presenting universal changes across all stages. The core characteristics can be summarized as “enrichment of pro-inflammatory/toxin-producing bacteria, depletion of beneficial bacteria, and overall reduced microbial diversity.

#### 4.1.1 Universal gut microbiota changes across CKD stages

Regardless of CKD stage, patients exhibit common imbalances of core phyla and key genera in the gut microbiota, which are the fundamental features of disease-related dysbiosis (38).At the phylum level, compared with healthy individuals, CKD patients have significantly increased abundances of Proteobacteria and Bacteroidetes in the gut, while the abundance of Firmicutes (rich in *Roseburia*, *Faecalibacterium*, etc.) is consistently reduced, directly leading to a significant reduction of the Firmicutes/Bacteroidetes (F/B) ratio (39–41). This ratio imbalance has been confirmed to be closely associated with increased intestinal barrier permeability, energy metabolism disorders, and systemic inflammatory status (42–44). In addition, there is no consistent trend in the overall abundance of Actinobacteria in the gut of ESRD patients, but the abundance of *Bifidobacterium*—a core beneficial genus within this phylum—is significantly decreased (45, 46), further impairing the anti-inflammatory and metabolic protective functions of the gut microbiota. This feature is particularly prominent in hemodialysis patients (45, 47).

At the genus level, the abundances of potential pathogenic bacteria—including Enterobacteriaceae and its subordinate genera *Escherichia-Shigella* and *Citrobacter*, as well as *Clostridium* and *Parabacteroides*—are significantly increased. In contrast, the abundances of beneficial bacterial families such as Lactobacillaceae and Lachnospiraceae, their subordinate genera (*Ruminococcus*, *Roseburia*, *Faecalibacterium*), and core SCFA-producing genera such as *Prevotella* are generally decreased (38, 39, 48). Among these, *Roseburia* and *Faecalibacterium*, as dominant beneficial genera in the gut of healthy individuals, show consistent depletion in CKD patients. The degree of their reduction is significantly negatively correlated with the decline in glomerular filtration rate (GFR), and they have been confirmed by multicenter studies as core signature genera of gut microbiota dysbiosis in CKD (39, 42, 49, 50).

At the functional level, the remodeling of microbial functional pathways is highly associated with the pathological progression of CKD (51). Functional modules related to “uremic toxin synthesis” (biosynthetic pathways of indoxyl sulfate [IS] and p-cresyl sulfate [p-CS]) and the “lipopolysaccharide (LPS) biosynthesis” pathway are significantly enriched, directly promoting enterogenic endotoxemia and uremic toxin accumulation (52). In contrast, protective pathways associated with “short-chain fatty acid (SCFA) synthesis” and “secondary bile acid biosynthesis” are significantly weakened, further impairing intestinal barrier protection, anti-inflammatory, and metabolic regulatory functions, and forming a vicious cycle of “structural dysbiosis - functional abnormalities” (53, 54).

#### 4.1.2 Stage-specific gut microbiota changes across different CKD stages

As CKD progresses from early stages (stages 1-2) to advanced stages (stages 4-5/ESRD), the gut microbiota exhibits distinct stage-specific remodeling characteristics in structure and function. Some genera are enriched or depleted only in specific stages, and these changes are closely associated with renal function indicators and pathological progression.

In early CKD (stages 1-2), a stage of mild renal impairment, specific microbial alterations have already occurred. Genera such as *Cetobacterium somerae* and *Candidatus Stoquefichus* sp. KLE1796 are specifically enriched, and the abundances of the phylum Tenericutes and class Mollicutes are significantly higher than those in healthy controls. Meanwhile, differences in the abundances of microbial genes related to secondary bile acid biosynthesis are observed, suggesting that bile acid metabolism disorders are initiated in early CKD (51). In addition, the abundance of *Megasphaera micronuciformis* increases at this stage, while the abundances of core SCFA-producing genera such as *Roseburia faecis* and *Bacteroides eggerthii* begin to decrease, serving as early signals of microbial functional abnormalities (51).

In mid-stage CKD (stage 3), with moderate renal function decline, microbiota dysbiosis further exacerbates. Potential pro-inflammatory genera such as *Fusobacterium mortiferum*, *Bariatricus massiliensis*, and *Bacteroides stercorirosoris* are specifically enriched, while the abundances of beneficial genera including *Alistipes indistinctus*, *Alistipes inops*, and *Bacteroides uniformis* are significantly reduced (42, 51). Notably, the abundance of *Parasutterella* starts to increase at this stage, and the correlation between its enrichment and the decline in estimated glomerular filtration rate (eGFR) gradually strengthens with disease progression, making it a mid-term warning marker for renal function deterioration (55).

In advanced CKD (stages 4-5/ESRD), the terminal stage of the disease, the gut microbiota structure undergoes significant remodeling. The abundances of genera such as *Merdibacter massiliensis*, *Clostridium glycyrrhizinilyticum*, and *Merdimonas faecis* are significantly elevated, while beneficial genera including *Prevotella* sp. 885 and *Weissella* confuse are further depleted (51). In ESRD patients, the abundance of *Turicibacter sanguinis* is increased, while the abundances of probiotic genera such as *Streptococcus mutans*, *Bifidobacterium adolescentis*, and *Lactobacillus crispatus* are decreased. Additionally, the abundances of the phylum Verrucomicrobia, genus *Akkermansia*, and genus *Blautia* show stage-specific elevation and reach their peaks (55). Among these, the abundance of *Akkermansia* is significantly positively correlated with blood urea nitrogen and serum creatinine (Scr) levels, and has been confirmed as a potential microbial biomarker for advanced CKD (51). Furthermore, the total intestinal bacterial load is reduced in ESRD patients, and the abundance of *Bacteroides* is significantly higher than that in healthy individuals. This abundance is positively correlated with the levels of uremic toxins IS and p-CS, directly linking toxin accumulation to aggravated renal injury (52, 56).

#### 4.1.3 Gut microbiota characteristics in dialysis patients

End-stage renal disease (ESRD) patients undergoing hemodialysis (HD) or peritoneal dialysis (PD) exhibit further exacerbated gut microbiota dysbiosis, which is closely associated with dialysis-related complications. The overall dysbiosis is characterized by persistent reduction in gut microbiota diversity. The abundances of potential pathogenic taxa, such as the phylum Proteobacteria and family Enterobacteriaceae, are significantly elevated, while the abundances of beneficial bacterial families including Euryarchaeota, Veillonellaceae, Lactobacillaceae, and Eubacteriaceae are further decreased—aggravating gut-kidney axis dysfunction (57, 58).

Regarding specific microbiota alterations, the gut of dialysis patients shows increased abundances of opportunistic pathogens (e.g., *Brachybacterium*, *Catenibacterium*) and bacteria from the families Halomonadaceae and Pseudomonadaceae. In contrast, the abundances of SCFA-producing taxa such as *Prevotella*ceae and the genus *Prevotella* are reduced, further impairing intestinal barrier function (52, 59).

**Figure 1:**
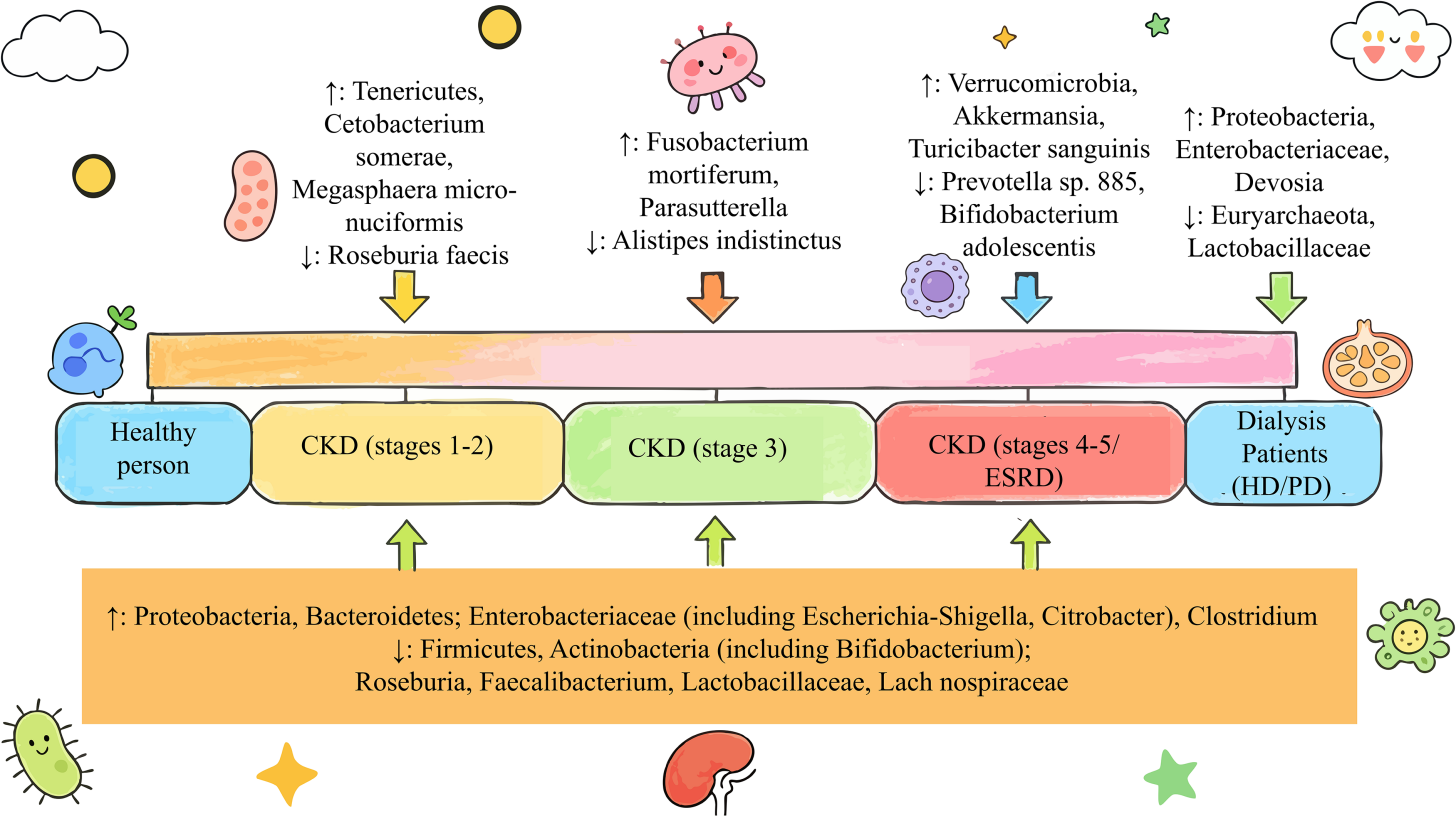
Dynamic changes in gut microbiota composition during chronic kidney disease (CKD) progression and dialysis stages. CKD, Chronic Kidney Disease; ESRD, End-Stage Renal Disease; HD, Hemodialysis; PD, Peritoneal Dialysis. This figure illustrates the differences in gut microbiota abundance among healthy individuals, patients with CKD at distinct stages (stages 1–2, stage 3, stages 4–5/end-stage renal disease [ESRD]), and patients undergoing maintenance dialysis (hemodialysis [HD]/peritoneal dialysis [PD]): CKD stages 1–2: Increased abundance of *Tenericutes, Cetobacterium somerae*, and *Megasphaera micronuciformis*, accompanied by decreased abundance of *Roseburia faecis*; CKD stage 3: Elevated abundance of *Fusobacterium mortiferum* and *Parasutterella*, with reduced abundance of *Alistipes indistinctus*; CKD stages 4–5/ESRD: Higher abundance of Verrucomicrobia, *Akkermansia*, and *Turicibacter sanguinis*, whereas *Prevotella* sp. 885 and Bifidobacterium adolescentis exhibit decreased abundance; Dialysis patients: Enrichment of Proteobacteria, Enterobacteriaceae, and Devosia, along with depletion of Euryarchaeota and Lactobacillaceae; Overall CKD trend (orange bar at the bottom): A general increase in the abundance of Proteobacteria, Bacteroidetes, Enterobacteriaceae (including *Escherichia-Shigella* and *Citrobacter*), and Clostridium; conversely, a reduction in the abundance of Firmicutes, Actinobacteria (including *Bifidobacterium*), *Roseburia, Faecalibacterium, Lactobacillaceae*, and *Lachnospiraceae*.

In terms of complication correlations, gut microbiota dysbiosis in PD patients is closely linked to gastrointestinal symptoms (e.g., dyspepsia, constipation). Additionally, the abundance of the genus *Devosia* in the blood is significantly positively correlated with vascular calcification—a key contributor to the high mortality rate in PD patients—suggesting that microbiota dysbiosis may participate in the development and progression of dialysis-related complications by mediating metabolic disorders (52). Furthermore, probiotic intervention can modulate the gut microbiota structure of PD patients and alleviate gastrointestinal symptoms, providing feasibility for microbiota-targeted interventions in this population (60) (**Figure 1**). 

## 5 Targeted links between the gut microbiome and bone diseases

### 5.1 Association between the gut microbiota and rheumatoid arthritis

Rheumatoid arthritis (RA) is an autoimmune disease characterized by chronic synovitis as the core pathological feature, accompanied by joint structure destruction and functional impairment. Its global disease burden has been continuously rising (61). According to data from the Global Burden of Disease (GBD) Study, the global number of RA cases reached 17.6 million in 2020, a 121% increase compared to 1990, and is projected to further rise to 31.7 million by 2050 (62, 63). Clinically, RA is mainly manifested as symmetric erosive polyarthritis; pathologically, it is centered on proliferative synovitis and progressive destruction of articular cartilage and bone tissue. It can also involve extra-articular systems such as the lungs, cardiovascular system, and nervous system, ultimately leading to severe dysfunction or even disability (64–66).

**Figure 2:**
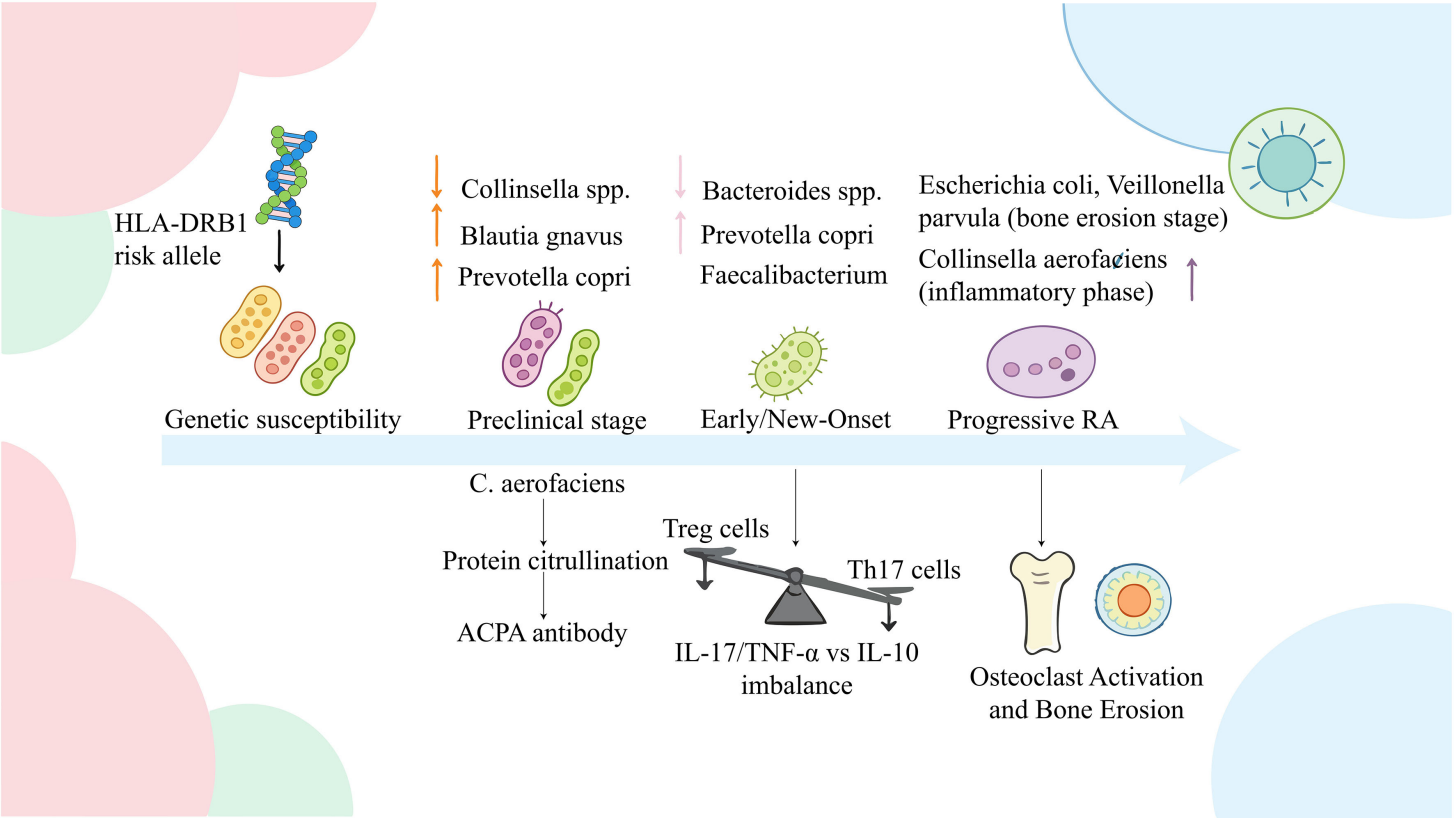
Schematic diagram of gut microbiota-driven pathogenic and immunometabolic mechanisms in rheumatoid arthritis (RA) across different disease stages; CKD, Chronic Kidney Disease ESRD, End-Stage Renal Disease. This figure illustrates the continuous progression of RA from genetic susceptibility to progressive bone erosion, highlighting the dynamic interactions between gut microbiota dysbiosis, immune imbalance, and pathological outcomes. In the genetic susceptibility stage, the HLA-DRB1 risk allele increases the host’s susceptibility to the disease. During the preclinical stage, the abundance of the genus *Collinsella* decreases, while the abundances of *Blautia gnavus* and *Prevotella* copri increase. Among these, *Collinsella* aerofaciens drives protein citrullination and promotes the production of anti-citrullinated protein antibodies (ACPA). In the early/new-onset RA stage, the abundance of *Bacteroides* decreases, while that of *Prevotella* copri increases and *Faecalibacterium* exhibits inconsistent changes. This stage is accompanied by an imbalance between regulatory T cells (Treg) and T helper 17 cells (Th17), leading to a shift in the cytokine profile toward pro-inflammatory IL-17/TNF-α and a decrease in the anti-inflammatory IL-10 level. In progressive RA, Escherichia coli, *Veillonella parvula* (bone erosion stage), and *Collinsella* aerofaciens (inflammatory stage) are enriched, ultimately resulting in osteoclast activation and bone erosion. HLA-DRB1, Human Leukocyte Antigen-DRB1 RA, Rheumatoid Arthritis C. aerofaciens, Collinsella aerofaciens (abbreviation of the species name) ACPA, Anti-Citrullinated Protein Antibody; Treg, Regulatory T Cell; Th17, T Helper 17 Cell; IL-17, Interleukin-17; TNF-α, Tumor Necrosis Factor-α; IL-10, Interleukin-10.

In recent years, as a key environmental regulator, the gut microbiota has gradually emerged as one of the core research directions in RA due to its important role in regulating host immune homeostasis and the pathogenesis of autoimmune diseases. The gut microbiota of RA patients exhibits significant dysbiosis throughout the preclinical stage, early diagnosis, and disease progression, with stage-specific remodeling characteristics. This dysbiosis even precedes the onset of typical joint symptoms and participates in disease initiation and progression (4, 67, 68) (**Figure 2**).

#### 5.1.1 Gut microbial characteristics of preclinical rheumatoid arthritis (high-risk population)

Gut microbial community instability has already emerged in the preclinical RA phase (serum anti-cyclic citrullinated peptide antibody ACPA-positive without typical joint symptoms), laying the foundation for the breakdown of immune tolerance in the disease. The most prominent feature of this phase is the significant enrichment of the Prevotellaceae family and its subordinate *Prevotella* genus—studies have shown that the detection rate of *Prevotella* in the gut of individuals with preclinical RA is significantly higher than that in the control group of first-degree relatives of RA patients, and changes in its abundance are dynamically associated with disease progression: in high-risk populations who are anti-CCP-positive and present with new-onset musculoskeletal symptoms, the abundance of *Prevotella* shows a fluctuating elevation as the time to RA diagnosis shortens, with a prominent manifestation during the gut microbial instability phase approximately 10 months before disease onset (69–72). Notably, *Prevotella* copri, a core species within this genus strongly associated with RA, has a detection rate of approximately 53% in individuals with preclinical RA and an even higher enrichment rate of up to 75% in patients with new-onset RA, both significantly higher than the 21.4% observed in healthy controls. These data suggest that *Prevotella copri* may serve as a key microbial biomarker for the early stage of RA pathogenesis (73, 74). In addition, the gut microbiota of patients with preclinical RA exhibits other characteristic alterations: the abundance of *Blautia gnavus* is significantly increased, while that of the *Collinsella* genus is decreased compared with healthy individuals (69–71). More importantly, a correlation exists between genetic susceptibility to RA and microbial composition—HLA-DRB1 RA risk alleles can directly modulate the overall structure of the gut microbiota, and RA-associated genetic variants are positively correlated with the abundance of *Prevotella* spp. This implies that genetic factors may indirectly disrupt immune tolerance and promote autoimmune activation by shaping the colonization characteristics of the gut microbiota, providing critical evidence for the genetic-microbial-immune axis in RA pathogenesis (68, 75).

Animal experiments have further validated these findings. The collagen-induced arthritis (CIA) model, a classic tool for investigating the pathogenesis of RA, exhibits significant gut microbial dysbiosis and metabolic abnormalities at 20 days after the initial collagen immunization—a preclinical stage prior to the onset of joint symptoms. At the microbial level, 20 genera including *Oscillospira*, *Bifidobacterium*, *Ruminococcus*, *Allobaculum*, and *Alistipes* show characteristic changes in abundance. At the metabolic level, 33 fecal metabolites display fluctuations, covering categories such as sugars and their derivatives, amino acids, long-chain fatty acids, and short-chain fatty acids (SCFAs), among which SCFA levels are strongly correlated with the abundance of genera including *Bifidobacterium*, *Alistipes*, and *Ruminococcus*. Notably, although mice show no joint symptoms at this stage, early activation of RA-related immune responses has already occurred, and the aforementioned microbial-metabolite dysbiosis is closely associated with the state of immune activation. These results provide direct experimental evidence for the early pathogenic mechanism of the gut microbial-metabolic-immune axis in RA (76).

However, inconsistencies exist in research conclusions regarding the abundance of Prevotellaceae in the preclinical RA phase. A previous large cohort study (SCREEN-RA, n=371) stratified first-degree relatives of RA patients into four preclinical subgroups: control, high genetic risk, autoimmune, and symptomatic. 16S rRNA sequencing revealed no significant differences in Prevotellaceae abundance among the subgroups, with no intergroup differences in fecal calprotectin levels either. This contradiction with previous conclusions of Prevotellaceae enrichment may be attributed to heterogeneity in study design, variations in sample characteristics, and differences in technical methodologies (77).

#### 5.1.2 Intestinal microbiota characteristics in early and new-onset rheumatoid arthritis

Patients with early rheumatoid arthritis (RA), particularly those with new-onset and untreated disease, exhibit the typical intestinal microbiota features of reduced diversity and community structural remodeling, which are closely correlated with the immunopathological progression of the disease. The specific manifestations are as follows: In terms of overall structural and phylum-genus level alterations, multiple cohort studies have confirmed that the alpha diversity of intestinal microbiota in patients with early RA is significantly lower than that in healthy individuals (78). A comparative study of 108 RA patients and 99 healthy controls revealed that at the phylum level, the intestinal microbiota of RA patients showed increased abundances of Actinobacteria and Proteobacteria, along with decreased abundances of Firmicutes, Fusobacteriota and Bacteroidota. At the genus level, the abundances of *Faecalibacterium*, *Blautia* and *Escherichia-Shigella* were elevated, while those of *Bacteroides* and *Coprococcus* were reduced; additionally, *Bifidobacterium* was identified as one of the most significantly differentially abundant genera in RA patients (79). Another study found that the relative abundance of *Prevotella copri* (*P. copri*) in the intestines of patients with new-onset RA was significantly higher than that in healthy controls, whereas the abundance of *Bacteroides* was markedly decreased. This pattern of *P. copri* enrichment and *Bacteroides* depletion may be involved in the initiation of early immune activation (78).

In terms of gender-specific characteristics, intestinal dysbiosis in female RA patients presents more distinct phenotypic specificity. Compared with healthy women, female RA patients had increased intestinal abundances of *Bacteroides*, *Megamonas* and *Oscillospira*, and reduced abundances of *Prevotella*, *Gemmiger* and *Roseburia*. Among these, the differential abundances of *Gemmiger*, *Bilophila* and *Odoribacter* can serve as potential diagnostic microbial biomarkers for female RA (80).

Regarding core microbiota and functional correlations, Huang et al. established the RA-derived Gut Microbiota Bank (RAGMB) using seven modified culture methods, and isolated 601 bacterial strains (encompassing 280 species, including 43 novel species) from fecal samples of 20 RA patients. This collection successfully covered 93.2% of the moderate-to-high abundance intestinal microbiota in RA and for the first time defined a core intestinal microbiota of RA consisting of 20 species. Among these, *Mediterraneibacter tenuis* and *Eubacterium rectale* were closely associated with clinical inflammatory markers: their abundances were positively correlated with the erythrocyte sedimentation rate (ESR) and negatively correlated with interleukin-10 (IL-10) levels. Further animal experiments confirmed that these two species could exacerbate host inflammatory responses by shortening colonic length, increasing spleen weight, reducing plasma IL-10 levels and elevating interleukin-17A (IL-17A) levels, which directly validates the regulatory effects of the core microbiota on RA immunopathology (4).

During the progression of RA, the intestinal microbiota exhibits distinct stage-specific dynamic evolutionary characteristics, with significant differences in microbial composition and metabolic functions across different pathological stages (inflammatory initiation, cartilage damage, bone erosion and late stage): In the inflammatory initiation stage, the core characteristic is a significant increase in the abundance of *Collinsella aerofaciens*, which is independently associated with high anti-cyclic citrullinated peptide antibody (ACPA) levels and smoking history in RA patients (81). Mechanistically, *C. aerofaciens* can mediate protein citrullination to generate citrullinated autoantigenic peptides by enhancing arginine deiminase (AD) activity (the gene detection rate of this enzyme in the intestines of RA patients is approximately 90%). These microbe-derived citrullinated antigens exhibit a molecular mimicry effect with endogenous human citrullinated proteins, which can directly trigger the immune system to produce ACPA, thereby initiating autoimmune responses and joint inflammation. This makes *C. aerofaciens* a key microbial driver of the initiation of RA immunopathology (82, 83).

In the cartilage damage stage, the core feature is the sustained decrease in the abundances of *Bacteroides uniformis* and *Bacteroides plebeius*. The depletion of these two genera directly leads to a significant impairment of the intestinal microbiota’s metabolic function of glycosaminoglycans (including dermatan sulfate and heparan sulfate). Glycosaminoglycans are key structural components of the articular cartilage matrix, and their metabolic disorders reduce the production of chondroitin 4-sulfate and continuously damage the structural integrity of articular cartilage, making these two genera key microbial drivers of cartilage damage throughout the entire course of RA (82).

In the bone erosion stage, there is a concomitant increase in the abundances of *Escherichia coli* and *Veillonella parvula*. *E. coli* is significantly associated with elevated rheumatoid factor (RF) levels by activating the argininosuccinate transferase pathway and the trans-cinnamic acid degradation pathway; it also depletes the anti-inflammatory L-arginine and promotes the release of pro-inflammatory cytokines such as IL-1β and TNF-α. *Veillonella parvula* shows a specific increase in abundance and can induce osteomyelitis. Together, these two species jointly activate bone resorption-related inflammatory pathways, exacerbating osteoclast hyperactivity and bone erosion (82, 84).

In the late stage, the persistent increase in intestinal barrier permeability induces the gut-joint microbial invasion axis. Validated in a cohort of 271 RA patients, gut-derived microbes (predominantly from the phyla Proteobacteria and Firmicutes), including *Eggerthella lenta*, *Bifidobacterium longum* and *Prevotella copri*, were detected in the synovial fluid; viable strains such as *Clostridium* sp*orogenes* and *Enterococcus gallinarum* were also successfully isolated. These microbes invade the joints through hematogenous or extracellular vesicle pathways, further exacerbating synovial inflammation and bone homeostasis imbalance, and forming a pathological vicious cycle (82, 84).

Furthermore, the stage-specific characteristics of the intestinal microbiota in RA patients have been further validated in cohort studies, and cross-site microbial correlations have been identified. The core genera exhibit dynamic changes across different disease stages: *Collinsella* spp. (especially *Collinsella* aerofaciens) are significantly enriched in patients with stage 1 RA, and this genus participates in early immune activation by increasing intestinal barrier permeability and inducing IL-17A expression. In contrast, patients with stage 4 RA show a specific increase in the abundances of *Bifidobacterium longum* and *Eggerthella lenta*, both of which are associated with intestinal barrier damage and subsequent microbial invasion of the joints (85). Meanwhile, cross-site microbiota analysis revealed shared microbial dysbiosis in the intestine, oral cavity and saliva of RA patients: *Lactobacillus salivarius* showed a significant increase in abundance in all three sites, with even higher levels during periods of high disease activity; in contrast, *Haemophilus* spp. were significantly depleted in all three sites, and their abundances were negatively correlated with serum autoantibody levels (86). These microbial characteristics can not only assist in the staging and activity assessment of RA but also provide insights into the microbial interaction mechanisms of the gut-oral-joint axis (**Table 1**). 

**Table 1:** Summary of clinical trials on changes in core gut microbiota in patients with rheumatoid arthritis.

Study population	Reference	Country/Region	Study type	Sample size	Detailed sample characteristics	Relative abundance changes of gut microbiota	
						(Increased)	(Decreased)
Patients with rheumatoid arthritis	(87)	South Korea	Observational Study	124	94 patients with rheumatoid arthritis and 30 healthy participants	*Blautia gnavus* (genus Blautia)	*Romboutsia*, *Collinsella*, *Bifidobacterium*, Clostridium sensu stricto 1, *Lactobacillus*
	(69)	Italy	Prospective Interventional Study	39	19 patients with rheumatoid arthritis and 20healthy participants	*Blautia gnavus* (genus Blautia)	Genus level: Acetanaerobacterium, Gracilibacter, *Prevotella*; Species level: *Acetanaerobacterium elongatum*, *Cellulomonas massiliensis*, *Gracilibacter thermotolerans*
	(88)	United Kingdom	Prospective Cross-sectional Comparative Study	69	Case group: 25 individuals with anti-CCP antibody positivity without clinical synovitis; Control group: 44 healthy individuals	Helicobacteraceae, Erysipelotrichaceae, Ruminococcaceae, Lachnospiraceae	Bacteroidaceae, Barnesiellaceae, Methanobacteriaceae
	(71)	South Korea	Cross-sectional Comparative Study	54	Control group: 25 healthy females; Case group: 29 females with early rheumatoid arthritis	Phylum level: Bacteroidetes; Class level: Bacteroidia; Order level: Bacteroidales; Genus level: *Prevotella*	Phylum level: *Actinobacteria*; Genus level: *Collinsella*, *Bifidobacterium*
	(89)	United States	Cross-sectional Comparative Study	114	New-onset untreated RA group: 44 cases; Chronic treated RA (CRA) group: 26 cases; Psoriatic arthritis (PsA) group: 16 cases; Control group: 28 healthy individuals	Prevotellaceae	Group XIV Clostridia, Lachnospiraceae, *Bacteroides*
	(90)	Italy	Cross-sectional Comparative Study	52	42 patients with rheumatoid arthritis; 10 healthy controls	Class level: Bacilli; Order level: Lactobacillales	*Faecalibacterium*, *Faecalibacterium prausnitzii*, *Flavobacterium*, Blautia coccoides
	(91)	China	Cross-sectional Comparative Study	148	128 patients with rheumatoid arthritis; 20 healthy subjects	Phylum level: Verrucomicrobiae, Gammaproteobacteria, Euryarchaeota (Class: Methanobacteria), Tenericutes (Class: Mollicutes); Order level: Clostridiales; Family level: Enterobacteriaceae; Genus level: *Akkermansia* (including *Akkermansia muciniphila*), *Collinsella* (including *Collinsella aerofaciens*), Blautia, *Klebsiella*	Genus level: *Bifidobacterium* (including *Bifidobacterium adolescentis* and *Bifidobacterium longum*), *Faecalibacterium* (including *Faecalibacterium prausnitzii*)
	(79)	China	Cross-sectional Comparative Study	207	108 patients with rheumatoid arthritis; 99 healthy subjects	Phylum level: *Actinobacteria*, Proteobacteria; Genus level: *Faecalibacterium*, Blautia, *Terrisporobacter*, *Escherichia-Shigella*, *Fusicatenibacter*, *Bifidobacterium* (phylum *Actinobacteria*), *Sutterella* (phylum Proteobacteria)	Phylum level: Firmicutes, Fusobacteriota, Bacteroidota; Family level: Bacteroidaceae, Marinifilaceae (phylum Bacteroidota); Genus level: *Bacteroides*, *Coprococcus*, *Parabacteroides*
	(92)	China	Cross-sectional Comparative Study	49	32 patients with rheumatoid arthritis; 17 healthy subjects	*Ruminococcus* 2	*Lachnospira*
	(93)	Egypt	Case-Control Study	60	45 patients with rheumatoid arthritis; 15 healthy subjects	Phylum level: Bacteroidetes, *Actinobacteria*, Proteobacteria	Phylum level: Firmicutes
	(94)	China	Cross-sectional Comparative Study	404	205 patients with rheumatoid arthritis; 199 healthy subjects	Phylum level: Proteobacteria; Family level: Hyphomicrobiaceae, Enterobacteriaceae, Halomonadaceae (phylum Proteobacteria); Genus level: *Escherichia*/*Shigella*, *Ruminococcus* 2, *Clostridium*_XVIII, *Clostridium*_XlVb, *Lactobacillus*	Phylum level: Firmicutes; Genus level: Lachnospiracea_incertae_sedis, *Prevotella*, *Clostridium*_XlVa, *Roseburia*, *Dialister*, Unclassified_Lachnospiraceae, Blautia, *Megamonas*, Unclassified_Clostridiales, *Gemmiger*, *Parasutterella*, *Acetivibrio*, *Coprococcus*, *Anaerostipes*
	(95)	France	Cross-sectional Comparative Study	35	17 patients with rheumatoid arthritis; 18 healthy subjects	Phylum level: Proteobacteria, Tenericutes, Synergistetes; Family level: Enterobacteriaceae, Desulfovibrionaceae, Succinivibrionaceae; Genus level: *Klebsiella*	Phylum level: Firmicutes, Bacteroidetes; Family level: Prevotellaceae, Paraprevotellaceae, Bifidobacteriaceae
	(96)	China	Multistage Validated Case-Control Study	52	26 patients with rheumatoid arthritis; 26 healthy subjects	Phylum level: Bacteroidetes, Proteobacteria, Verrucomicrobia; Genus level: *Klebsiella*, *Enterococcus*, *Eisenbergiella*, *Escherichia*, *Flavobacterium*	Phylum level: Firmicutes, *Actinobacteria*; Genus level: Clostridiales_unclassified, *Megamonas*, *Fusicatenibacter*
	(97)	Japan	Case-Control Study	124	82 patients with rheumatoid arthritis; 42 healthy subjects	*Prevotella* (including *P. denticola*, *P. marshii*, P. disiens, P. corporis, P. amnii)	-
	(98)	Brazil	Case-Control Study	50	20 patients with rheumatoid arthritis; 30 healthy subjects	*Bacteroides*, *Prevotella*	*Clostridium leptum*
	(99)	United States	Case-Control Study	72	40 patients with rheumatoid arthritis; 32 healthy subjects	Phylum level: *Actinobacteria*; Class level: Bacilli (phylum Firmicutes); Order level: Clostridiales (phylum Firmicutes); Genus level: Eggerthella, Actinomyces, *Collinsella* (phylum *Actinobacteria*), *Eubacterium* (order Clostridiales), Turicibacter, Streptococcus (class Bacilli)	*Faecalibacterium*
	(81)	Spain	Cross-sectional Study	80	40 patients with rheumatoid arthritis; 40 healthy subjects	Family level: Enterococcaceae, Comamonadaceae, Moraxellaceae, Eubacteriaceae; Genus level: *Enterococcus*, Sedimentibacter, *Collinsella*; Species level: Collinsella aerofaciens	Genus level: *Sarcina*, 02d06, *Porphyromonas*; Species level: *Dorea formicigenerans*
	(100)	Spain	Cross-sectional Study	220	110 patients with rheumatoid arthritis; 110 healthy subjects	Phylum level: *Actinobacteria*, Spirochaetes, Synergistetes; Family level: *Coriobacteriaceae*, EtOH8 family (phylum *Actinobacteria*); Genus level: *Collinsella*, *Bifidobacterium* (phylum *Actinobacteria*), *Synergistes* (phylum Synergistetes), Unidentified genus of EtOH8 family, Unidentified genus of TM7-3 class, rc4-4, *Succinatimonas*, *Eubacterium*, *Anaerotruncus*, *Mogibacterium*, *Weissella*	Phylum level: Firmicutes; Family level: Streptococcaceae (phylum Firmicutes), Oxalobacteraceae, Unidentified family of order RF32 (enriched in healthy controls); Genus level: *Veillonella*, Dorea, *Coprococcus*, Unidentified genus of Lachnospiraceae, Unidentified genus of Erysipelotrichaceae (in RA patients with moderate/high disease activity)
	(86)	China	Cross-sectional Study	157	77 patients with rheumatoid arthritis; 80 healthy subjects	*L. salivarius*	*Haemophilus* *K. pneumoniae*, *B. bifidum*, *S. wadsworthensis*, *M. hypermegale*
	(101)	China	Cross-sectional Study	71	42 patients with rheumatoid arthritis; 29 healthy subjects	Fungal genera: *Wallemia*, *Phanerochaete*, *Suhomyces*, *Tolypocladium*, *Trebouxia, Tremella, Scolecostigmina, Sarocladium; Candida*	Fungal genera: *Pholiota*, *Scedosporium, Trichosporon, Ganoderma, Auricularia, Aureobasidium, Pleurotus, Podosphaera, Dipodascus, Alternaria, Dendroclathra, Phacidium, Septobasidium*
	(102)	South Korea	Cross-sectional Study	129	99 patients with rheumatoid arthritis; 30 healthy subjects	Class level: Saccharomycetes; Genus level: Candida; Genera with trending increased abundance: *Kazachstania, Issatchenkia, Penicillium, Mucor; LEfSe-selected genera: Meyerozyma, Aureobasidium, Xeromyces, Coprinopsis, Wallemia*	Phylum level: Mucoromycota; Family level: Aspergillaceae; Genus level: *Aspergillus*; LEfSe-selected genera: *Conocybe*, *Monascus*, *Schizosaccharomyces*
	(80)	China	Cross-sectional Comparative Study	47	40 females with rheumatoid arthritis; 7 healthy females	Phylum level: Proteobacteria, Fusobacteria; Genus level: *Bacteroides*, *Megamonas*, Oscillospira, Lactococcus, Butyricicoccus, Epulopiscium, Megasphaera, Desulfovibrio	Phylum level: Firmicutes, Bacteroidetes; Genus level: *Prevotella*, *Faecalibacterium*, *Roseburia*, *Sutterella*, *Gemmiger*, *Parabacteroides*, *Alistipes*, *Bifidobacterium*, *Barnesiella*
	(103)	Kazakhstan	Observational Case-Control Study	190	77 females with rheumatoid arthritis; 113 healthy females	Family level: Lachnospiraceae; Genus level: Dorea, Sellimonas, Tyzzerella, Lachnospiraceae_NK4A136_group (family Lachnospiraceae)	Phylum level: Proteobacteria, Actinobacteriota; Family level: Oscillospiraceae; Genus level: NK4A214 group (family Oscillospiraceae); Additionally, enriched in healthy controls: Staphylococcaceae, *Coriobacteriaceae*, Rhizobiaceae, Sphingomonadaceae, Puniceicoccaceae, Eubacterium Coprostanoligenes group
	(104)	China	Cross-sectional Study	91	High-risk pre-RA group: 53 individuals; Healthy control group: 38 individuals	Genus level: *Lactobacillus*, *Raoultibacter*, Eubacterium_brachy_group, *Enorma*, *Holdemania*	Genus level: *Ruminococcus*_2, *Pseudomonas*, *Ruminiclostridium*_5, *Coprococcus*_2, Ruminococcaceae_UCG-009, *Chryseobacterium*
	(105)	China	Cross-sectional Study	464	195 patients with rheumatoid arthritis; 269 healthy subjects	Genus level: Eubacterium_hallii_group, *Escherichia-Shigella*, Streptococcus	Genus level: *Megamonas*, *Bacteroides*, *Faecalibacterium*
	(70)	Switzerland	Cross-sectional Study	133	83 patients with rheumatoid arthritis; 50 healthy subjects	Prevotellaceae family, particularly Prevotella spp.	-
	(92)	China	Cross-sectional Study	49	32 patients with rheumatoid arthritis; 17 healthy subjects	*Ruminococcus* 2	*Lachnospira*
	(106)	China	Cross-sectional Study	737	262 patients with rheumatoid arthritis; 475 healthy subjects	Self-test data: Phylum: Bacteroidetes, Proteobacteria, Verrucomicrobia; Genus: *Bacteroides*, *Shigella*, *Ruminococcus*_gnavus_group; PRJNA450340 dataset: Phylum: *Actinobacteria*, Verrucomicrobia, Euryarchaeota; Genus: Blautia; LEfSe analysis: *Ruminococcus*_gnavus_group	Self-test data: Phylum: Firmicutes; Genus: Blautia; PRJNA450340 dataset: Phylum: Firmicutes, Bacteroidota; Genus: *Romboutsia*, *Subdoligranulum*; LEfSe analysis: *Fusicatenibacter*, Butyricicoccus, *Subdoligranulum*, Erysipelotrichaceae_UCG-003, *Romboutsia*, Dorea (HC-enriched)
	(107)	China	Cross-sectional Study	75	50 patients with rheumatoid arthritis; 25 healthy subjects	Firmicutes	Phylum level: Acidobacteria; Genus level: Lachnospiraceae_Clostridium, *Megamonas*
	(108)	Iran	Cross-sectional Study	50	25 patients with rheumatoid arthritis; 25 healthy subjects	-	*Bacteroides fragilis*, *Roseburia faecis*, *Fusobacterium nucleatum*
	(109)	China	Cross-sectional Study	266	164 patients with rheumatoid arthritis; 102 healthy subjects	-	Phycodnaviridae (algal DNA virus family)
类风湿关节炎患者	(82)	China	Cross-sectional Study	122	76 patients with rheumatoid arthritis (RA), 19 patients with osteoarthritis (OA), 27 healthy individuals	Phylum level: Firmicutes, *Actinobacteria*; Species level: Bifidobacterium dentium, Collinsella aerofaciens, Veillonella parvula, Eggerthella lenta, Bifidobacterium longum, Dialister invisus, *Escherichia coli*	Phylum level: Bacteroidetes; Species level: Bacteroides uniformis, Bacteroides plebeius
	(4)	China	Experimental Study	20	Fecal samples from 20 RA patients	Genus level: Mediterraneibacter tenuis, Bacteroides vulgatus, *Bacteroides ovatus*, *Escherichia coli*; Species level: *Escherichia coli*	Genus level: Eubacterium rectale; Species level: *Anaerostipes hadrus*, *Blautia longa*, *Faecalibacterium prausnitzii*, Fusicatenibacter sacchanivorans, *Parabacteroides merdae*, *Viscinimonas butyriproducens*, uncultured *Subdoligranulum*_sp; Repeated species: *Anaerostipes hadrus*, *Blautia longa*, Fusicatenibacter sacchanivorans, uncultured *Subdoligranulum*_sp
	(110)	China	Cross-sectional Comparative Study	59	29 patients with rheumatoid arthritis; 30 healthy subjects	Phylum level: Proteobacteria, Verrucomicrobia; Class level: c_Verrucomicrobiae, c_Bacilli; Order level: o_Verrucomicrobiales, o_Lactobacillales; Family level: f_Streptococcaceae, f_Akkermansiaceae; Genus level: Streptococcus, *Akkermansia*, g_Blautia, g_*Lactobacillus*, g_Eggerthella; Species level: s_uncultured_bacterium_g_Blautia, s_uncultured_bacterium_g_*Akkermansia*, s_uncultured_bacterium_g_Streptococcus	Phylum level: Bacteroidetes, Cyanobacteria, Synergistetes; Class level: c_Bacteroidia; Order level: o_Bacteroidales; Family level: f_Ruminococcaceae, f_Prevotellaceae; Genus level: Faecalibacterium, g_*Bacteroides*, g_*Prevotella*, g_*Bifidobacterium*, g_*Ruminococcus*_1, g_Ruminococcaceae_UCG-002, g_Lachnospiraceae_UCG-001, g_Fusobacterium, g_uncultured_bacterium_f_Prevotellaceae; Species level: s_uncultured_bacterium_g_*Faecalibacterium*
	(111)	China	Cross-sectional Study	80	60 patients with rheumatoid arthritis; 20 healthy subjects	Blautia spp., Streptococcus spp.	*Roseburia* spp., Lachnoclostridium spp.
	(112)	China	Cross-sectional Comparative Study	126	66 patients with rheumatoid arthritis; 60 healthy controls	Phylum level: Bacteroidetes; Genus level: *Bacteroides*, *Escherichia-Shigella*, *Parasutterella*, Flavonifractor, Eubacterium xylanophium group, Tyzzerella, Sellimonas, Oscillospira, Eubacterium saphenum group	Phylum level: Firmicutes, Proteobacteria, *Actinobacteria*; Genus level: *Lactobacillus*, *Alloprevotella*, *Enterobacter*, Clostridium sensu stricto-3, Ruminococcaceae UCG-014, *Odoribacter*, Rikenellaceae RC9, *Enterococcus*, *Klebsiella*, Desulfovibrio, Citrobacter, *Akkermansia*, Helicobacter, *Rikenella*, *Staphylococcus*, *Coprococcus* 1, *Coriobacteriaceae* UCG-002, *Rhodococcus*

### 5.2 Specific dysbiosis characteristics of intestinal microbiota associated with osteoarthritis

Osteoarthritis (OA) is a common chronic degenerative joint disease characterized by the core pathological features of progressive degeneration of articular cartilage, osteophyte formation, and synovial inflammation (present or absent), with clinical manifestations of progressive joint pain and dysfunction (113), Its global disease burden has shown a marked upward trend—relevant studies on global early-onset OA have revealed that the number of new cases, prevalent cases, and Years Lived with Disability (YLDs) related to early-onset OA worldwide all doubled from 1990 to 2019, and 98.04% of countries exhibited a continuous upward trend in age-standardized incidence rates, prevalence rates, and YLDs rates of the disease. As of 2019, 52.31% of new OA cases worldwide were early-onset cases, and this disease has imposed a substantial health and economic burden globally: the associated healthcare expenditure alone reached 46.17 billion US dollars in 2019, with productivity losses amounting to 60.70 billion US dollars (114).

**Figure 3:**
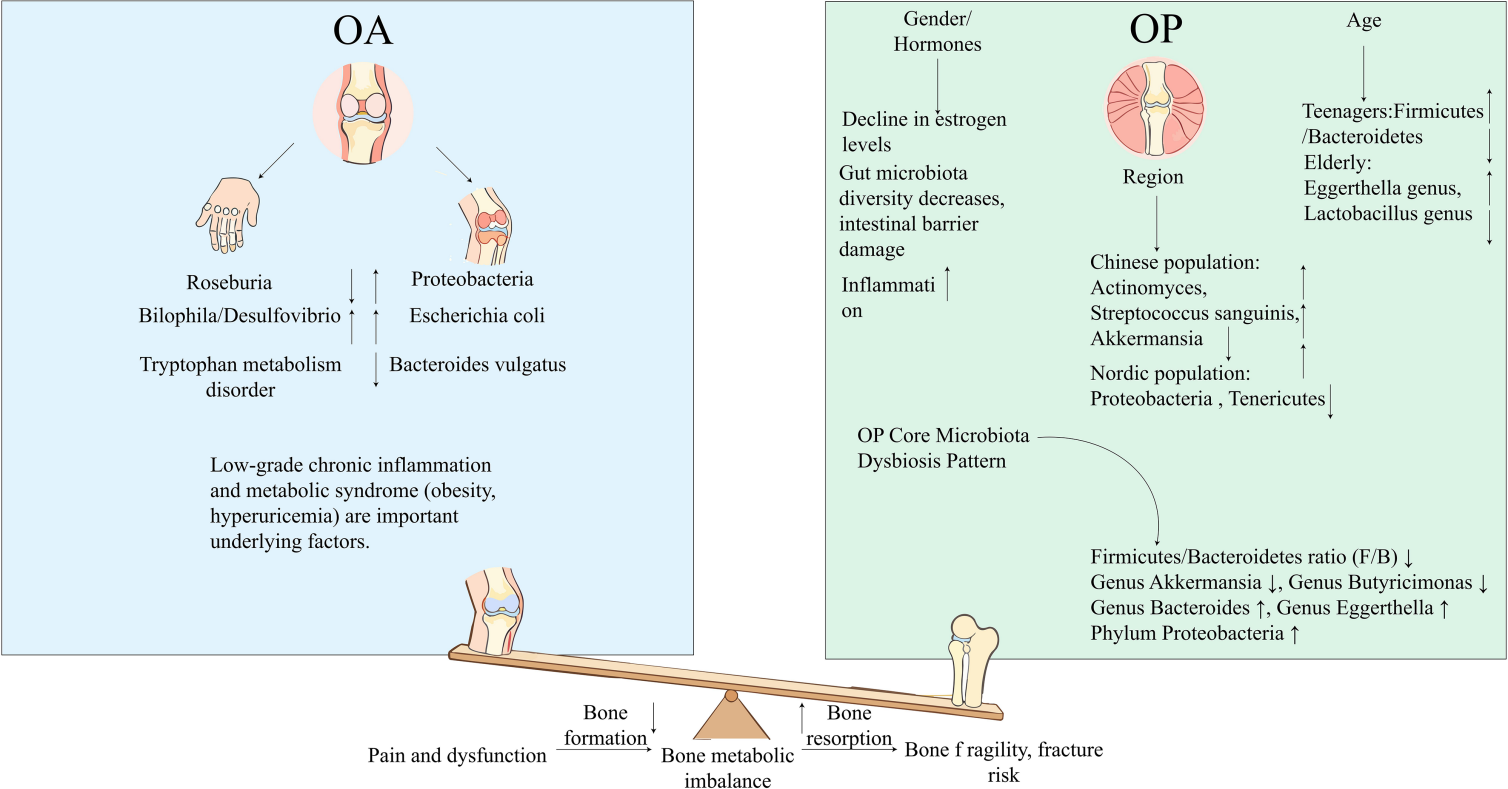
Schematic diagram of gut microbiota-mediated bone metabolic imbalance in osteoarthritis (OA) and osteoporosis (OP). This figure employs a seesaw model to compare the distinct mechanisms through which intestinal dysbiosis drives bone metabolic imbalance in osteoarthritis (OA) and osteoporosis (OP). On the left (OA, light blue area), the disease is associated with decreased abundance of *Roseburia*, increased abundance of *Bilophila*/*Desulfovibrio* and the phylum Proteobacteria (including *Escherichia coli*), reduced levels of *Bacteroides vulgatus*, and tryptophan metabolic dysregulation. Low-grade chronic inflammation and metabolic syndrome act as the core underlying factors, ultimately leading to impaired bone formation, as well as joint pain and dysfunction. On the right (OP, light green area), the pathogenesis is linked to decreased estrogen levels and age- and region-specific alterations in the intestinal microbiota. The core pattern of intestinal dysbiosis in OP is characterized by a reduced Firmicutes/Bacteroidetes (F/B) ratio, decreased abundance of *Akkermansia* and *Butyricimonas*, and increased abundance of *Bacteroides*, *Eggerthella* and the phylum Proteobacteria. These microbial changes promote increased bone resorption, elevated bone fragility and a heightened risk of fracture. The central seesaw visually illustrates how OA and OP, via the two opposite bone metabolic alterations of reduced bone formation and increased bone resorption, collectively form a comprehensive landscape of bone metabolic imbalance. OA, Osteoarthritis; OP, Osteoporosis; F/B, Firmicutes/Bacteroidetes ratio.

OA has traditionally been classified as a degenerative joint disease, but recent research has subverted this traditional notion—inflammation has been clearly identified as one of its key pathological processes, and low-grade chronic inflammation plays a central role in the initiation and progression of the disease (115). Meanwhile, evidence supporting the association between intestinal dysbiosis and OA has become increasingly compelling: both animal experiments and clinical association studies have confirmed the presence of structural and functional abnormalities in the intestinal microbiota of OA patients and animal models. Yan Y et al. found that the intestinal microbiota diversity was reduced in rhesus monkey OA models, with altered abundances of genera including Mollicutes and *Lactobacillus*; additionally, microbial metabolic pathways were directly associated with cartilage damage. Intestinal dysbiosis may participate in the pathological regulation of OA by regulating low-grade inflammatory responses, influencing metabolic syndrome or inflammaging, and through other related pathways (116, 117) (**Figure 3**). 

#### 5.2.1 Alterations in the overall gut microbiota composition

The intestinal microbiota of patients with osteoarthritis (OA) exhibits significant dysbiosis across the phylum, family, genus, and species levels (118). The overall characteristics of intestinal microbiota in OA patients are as follows: at the phylum level, the compositional ratios of Firmicutes, Bacteroidota/Bacteroidetes, Proteobacteria, and Actinobacteria are imbalanced. Several studies have shown an increased abundance of Bacteroidota in OA cohorts, with elevated levels of pro-inflammatory taxa (genus *Clostridium*) and reduced abundances of beneficial taxa (genera *Agathobacter* and *Roseburia*) within Firmicutes (119, 120). Additionally, other research has indicated that Proteobacteria is the dominant phylum in the gut of patients with knee OA, and its increased abundance may be associated with intestinal barrier disruption and endotoxin translocation (121). Notably, study heterogeneity exists in the alterations of Bacteroidota: while some cohorts have demonstrated overall enrichment of Bacteroidota in OA patients, species-level analysis revealed a significant reduction in the abundances of beneficial species such as *Bacteroides stercoris* and *Bacteroides vulgatus*, reflecting the complexity of microbial characteristics at the phylum versus species levels. At the genus level, such dysbiosis is more specific: the abundances of multiple pro-inflammatory or potential pathogenic genera including *Clostridium*, *Streptococcus*, *Bacteroides*, and *Klebsiella* are elevated (118, 122).

In contrast, the abundances of commensal genera with anti-inflammatory properties or the capacity to produce beneficial metabolites (e.g., butyrate)—represented by *Roseburia*, *Faecalibacterium*, *Lactobacillus*, and *Bifidobacterium*—are universally decreased (119, 123).

At the species level, OA patients show a marked increase in the abundances of potential pro-inflammatory pathogenic bacteria such as *Escherichia coli*, *Klebsiella pneumoniae*, *Shigella flexneri*, and *Streptococcus salivarius*; conversely, the abundances of beneficial bacteria with metabolic protective or anti-inflammatory effects (e.g., *Bacteroides vulgatus*, *Bacteroides stercoris*, *Bacteroides uniformis*, *Bifidobacterium longum*, and *Prevotella copri*) are significantly reduced (121, 123, 124). Furthermore, study heterogeneity is observed in the diversity characteristics of the intestinal microbiota in OA patients: regarding alpha diversity, some studies have reported a significant decrease in microbial richness and diversity in elderly female OA patients, whereas no notable differences in alpha diversity were detected in other studies. In contrast, beta diversity consistently exhibits significant alterations across all studies, indicating that the core of OA-associated intestinal dysbiosis lies in the aberrant remodeling of the microbial community composition and structure, rather than a simple change in species richness (6, 119, 123).

#### 5.2.2 Site-specific intestinal microbiota characteristics of OA with different onset locations

Patients with symptomatic or erosive hand OA present with unique microbial and metabolic dysregulation. The Xiangya Osteoarthritis Study, which enrolled 1,388 community-dwelling middle-aged and elderly individuals, found a significant alteration in the beta diversity of intestinal microbiota in patients with symptomatic hand OA, accompanied by elevated abundances of hydrogen sulfide-producing potential pro-inflammatory genera *Bilophila* and *Desulfovibrio*, and a reduced abundance of the butyrate-producing anti-inflammatory genus *Roseburia* (122).

The DIGICOD cohort (416 patients with hand OA) confirmed the dysregulation of the tryptophan metabolic pathway in erosive hand OA: decreased levels of tryptophan and indole-3-aldehyde, increased levels of 5-hydroxytryptophan (5-OH-tryptophan) and 3-hydroxyanthranilic acid, and upregulation of the pro-inflammatory indoleamine 2,3-dioxygenase (IDO) pathway. Moreover, these metabolites were closely associated with pain phenotypes (e.g., serotonin levels were negatively correlated with the number of tender joints, and 3-hydroxykynurenine levels were positively correlated with AUSCAN pain scores) (125). Obese patients with combined hand and knee OA exhibited elevated proteolytic products in the fecal metabolome, along with dysregulated leukotriene and tryptophan metabolic pathways, suggesting that microbiota-mediated metabolic abnormalities may regulate disease progression through inflammatory pathways (126).

As the most common subtype of OA, knee OA is characterized by distinct specific alterations in intestinal microbiota. The Rotterdam Study (N = 1,427) and the Lifelines-DEEP Study (867 Caucasian adults) verified a significant correlation between the intestinal abundance of *Streptococcus* and the severity of joint pain in knee OA. This association is driven by local inflammation, and it is hypothesized that microbial endotoxins activate the inflammatory response of synovial macrophages to promote pain progression, providing evidence for intestinal microbiota as a potential therapeutic target for pain in knee OA (127, 128).

16S rRNA gene sequencing of patients with knee OA revealed that Proteobacteria is one of the dominant phyla, with increased abundances of *Prevotella_7*, *Clostridium*, and *Flavonifractor*, and decreased abundances of Agathobacter, *Ruminococcus*, and *Roseburia*. Metagenomic sequencing identified elevated abundances of pathogenic bacteria such as *Escherichia coli* and the genus *Peptococcus*, and a reduced abundance of beneficial bacteria such as *Bacteroides vulgatus* in knee OA patients. Additionally, the OA cohort showed significant alterations in microbial beta diversity and enrichment of Bacteroidota, while the healthy control group had a higher abundance of *Prevotella copri*, which exhibited good diagnostic efficacy for OA (6, 121, 129) (**Table 2**).

**Table 2:** Summary of clinical trials on changes in core gut microbiota in patients with osteoarthritis.

Reference	Country/Region	Study type	Sample size	Detailed sample characteristics	Relative abundance changes of gut microbiota	
					[Increased]	[Decreased]
(122)	China	Longitudinal Cohort Study	1388	Participants with symptomatic hand osteoarthritis [n=72] vs. participants with asymptomatic hand OA [n=1,316]	Family level: Christensenellaceae, Desulfovibrionaceae, Mogibacteriaceae; Genus level: *Bilophila*, *Desulfovibrio*	Family level: Lachnospiraceae; Genus level: Roseburia
(118)	China	Cross-sectional Study	200	20 healthy volunteers; 180 patients with knee osteoarthritis [with osteophyte formation]	Phylum level: Actinobacteria, Actinobacteriota; Genus level: *Blautia*, *Granulicatella*, *Bifidobacterium*, *Fusobacterium*	Genus level: *Phascolarctobacterium*
(123)	China	Case-Control Study	90	44 patients with osteoarthritis; 46 healthy controls	Phylum level: Actinobacteriota, Proteobacteria; Genus level: *Anaerostipes*, Bifidobacterium, *Brachyspira*, *Eggerthella*, etc.; Species level: *Anaerostipes hadrus*, *Prevotella sp900313215, Eubacterium_E hallii A*, *Bifidobacterium sp002742445*, Anaerostipes hadrus A, Blautia A, *Catenibacterium* sp000437715, *Bifidobacterium dentium*	Phylum level: Firmicutes; Genus level: *Faecalibacterium*, *Lachnoclostridium*, *Phascolarctobacterium*, *Paraprevotella*; Species level: *Bacteroides plebeius A*, *Roseburia inulinivorans*, *Dialister sp900343095*, *Phascolarctobacterium faecium*, multiple subtypes of *Faecalibacterium prausnitzii* [G, K, C, E], *Prevotella stercorea*, *Prevotella sp003447235*, *Prevotella copri A*
(130)	China	Case-Control Study	207	87 patients with knee synovitis; 120 healthy controls	*Phylum level: Actinobacteriota; Species level: Significantly increased ratio of Ruminococcus gnavus to Bacteroides stercoris*	*Phylum level: Bacteroidetes; Species level: Bacteroides stercoris*
(131)	China	Prospective Cohort Study	1324	46 cases of erosive hand osteoarthritis; 333 cases of non-erosive hand osteoarthritis; 945 healthy controls	*Citrobacter koseri, Fournierella massiliensis, Coprococcus eutactus A, Clostridium citroniae, Alistipes senegalensis*	*-*
(132)	China	Case-Control Study	38	23 patients with osteoarthritis; 15 healthy controls	Genus level: *Acidaminococcus*, *Gordonibacter*, *Exiguobacterium*, *Lachnoclostridium*; Species level: *Parabacteroides sp. CT06*, *Romboutsia ilealis, Butyrivibrio crossotus*, Bacteroidaceae bacterium DJF B220	*Genus level: Ruminococcus, Faecalibacterium; Species level: Bacteroides plebeius, Faecalibacterium prausnitzii, Bacteroides coprocola*
(133)	China	Observational Study	1359	70 patients with hand osteoarthritis; 1289 healthy controls	*Bilophila wadsworthia*, *Lactobacillus mucosae*, *Citrobacter koseri*, *Hungatella hathewayi*	*Roseburia intestinalis*, *Bacteroides spp*., *Haemophilus spp*.
(121)	China	Case-Control Study	89	32 patients with osteoarthritis; 57 healthy controls	Phylum level: Proteobacteria; Genus level: *Escherichia_Shigella*, *Prevotella*_7, *Clostridium*, Flavonifractor, *Klebsiella*, *BurkholderiaCaballeroniaParaburkholderia*, *Ruminococcus*_gnavus_group; Species level: *Escherichia coli*, *Klebsiella pneumoniae*, *Shigella flexneri*, *Streptococcus salivarius*	Genus level: *Agathobacter*, *Ruminococcus*, Roseburia, *Subdoligranulum*, *Lactobacillus*, *Coprococcus*_2; Family level: Lactobacillaceae, Christensenellaceae, Clostridiaceae_1, Acidaminococcaceae [enriched in healthy controls]; Species level: *Bacteroides vulgatus*, *Bacteroides stercoris*, *Bacteroides uniformis*
(6)	China	Case-Control Study	80	40 patients with osteoarthritis; 40 healthy controls	Phylum level: Bacteroidota, Verrucomicrobiota; Class level: Bacteroidia, Verrucomicrobiae; Order level: Bacteroidales, Verrucomicrobiales; Family level: Bacteroidaceae, Akkermansiaceae; Genus level: *Bacteroides*, *Akkermansia*; Species level: *Prevotella copri*	Phylum level: Firmicutes, Actinobacteriota; Class level: Clostridia, Actinobacteria, Negativicutes; Order level: Oscillospirales, Veillonellales-Selenomonadales, Bifidobacteriales; Family level: Ruminococcaceae, Veillonellaceae, Bifidobacteriaceae; Genus level: *Faecalibacterium*, Bifidobacterium, Dialister, *Megasphaera*; Species level: *Dialister sp*.

### 5.3 Osteoporosis-associated intestinal microbiota imbalance characteristics and population heterogeneity

Osteoporosis (OP) is a systemic metabolic bone disease characterized by reduced bone mass, disrupted bone microstructure, and a markedly elevated risk of fragility fractures. Its global epidemic has become increasingly severe, emerging as a major public health challenge (134, 135). Studies have shown that the number of confirmed OP cases worldwide has doubled compared with previous figures and is projected to further rise to 263 million between 2030 and 2034 (136). According to data from the World Health Organization (WHO), approximately 415 million people worldwide are currently affected by OP-related bone health issues, with 8.9 million fragility fractures caused by OP each year. Therefore, strengthening the early prevention and standardized management of OP holds great public health significance for alleviating the global medical burden and improving population health and quality of life (137).

The pathological essence of OP is bone metabolic imbalance. In normal bone remodeling, bone formation and bone resorption are dynamically coordinated via Basic Multicellular Units (BMUs). In the OP state, however, osteoclast activity is enhanced and bone resorption increased, while osteoblast function is impaired and bone formation reduced; additionally, the secretory factors of bone cells (osteoclasts, osteoblasts, and osteocytes) are aberrant. For instance, activated osteoclasts dissolve bone minerals and degrade bone tissue by secreting cathepsin K, Matrix Metalloproteinase 9 (MMP-9), and hydrochloric acid; osteocytes release sclerostin and other factors to inhibit bone formation; and Receptor Activator of Nuclear Factor-κB Ligand (RANKL) secreted by osteoblasts and osteocytes can activate the RANK-RANKL interaction and promote the expression of genes associated with osteoclastogenesis (134, 138).

In recent years, studies have found that intestinal dysbiosis is prevalent in OP patients, with its core characteristic being a significant reduction in microbial community diversity (139). Despite variations across different studies and populations, several consistent trends have been observed: at the phylum level, the intestinal microbiota of individuals with OP or low bone mineral density (BMD) typically exhibits a decreased Firmicutes/Bacteroidetes (F/B) ratio. Notably, the correlation between this ratio and bone mass is research context-dependent: in human populations with low BMD, the F/B ratio is positively correlated with BMD, whereas in animal models of high-fat diet-induced bone loss, the F/B ratio is negatively correlated with bone volume. Overall, a reduced F/B ratio is one of the characteristic microbial alterations in humans with OP or low BMD (5, 140).

In addition, the enrichment of the phylum Proteobacteria is closely linked to abnormal bone health (141). Animal experiments and clinical association studies suggest that an increase in Proteobacteria is often accompanied by intestinal dysbiosis and enhanced inflammatory responses. It may be indirectly associated with reduced bone mass or impaired bone mechanical properties by promoting the release of pro-inflammatory cytokines (e.g., TNF-α, IL-6) and altering the material properties of bone tissue, thus serving as a potential microbial biomarker for bone metabolic disorders (139, 142).

At the genus level, the intestinal microbiota of OP patients presents a characteristic imbalance: the absolute or relative abundances of *Bacteroides*, *Eggerthella*, Eisenbergiella, and *Clostridium_XlVa* are significantly elevated. In contrast, the abundances of beneficial genera with intestinal barrier-protective and anti-inflammatory properties are markedly reduced, including *Akkermansia*, *Butyricimonas*, and butyrate-producing genera (e.g., *Lactobacillus*) (143). Among these, the elevated abundance of *Bacteroides* is also significantly associated with decreased lumbar spine BMD and T-scores, while the enrichment of *Akkermansia* and *Butyricimonas* correlates with elevated bone formation markers and reduced bone resorption markers. These findings imply that such genera may contribute to the pathogenesis of OP by regulating bone metabolic homeostasis (144, 145).

#### 5.3.1 Population heterogeneity characteristics of OP-associated intestinal microbiota

The heterogeneity in the intestinal microbiota composition of OP patients is closely linked to age, geographical region, and sex, with age being the core regulatory variable. In terms of age, elderly OP patients are characterized by the enrichment of *Eggerthella* and *Clostridium*_XIVa and the reduction of beneficial genera such as *Lactobacillus* and *Veillonella*; this microbial profile may disrupt bone metabolism by exacerbating inflammation. Although the dominant phyla remain Bacteroidota and Firmicutes in the adolescent population with low BMD/OP, this group exhibits a decrease in Bacteroidota and an increase in Firmicutes, which correlates with BMD reduction. These observations highlight the deterministic effect of age on the microbiota-bone metabolism association (146, 147). In the elderly, age-related immunosenescence and alterations in intestinal microbiota composition may also alter the bioactivity of metabolites, converting bone-protective metabolites into bone catabolic mediators and thus accelerating bone resorption and OP progression (148, 149).

Regarding geographical distribution, the Nordic population (Finnish cohort) shows an elevation in Proteobacteria and a reduction in Tenericutes, which is associated with OP/fracture risk (150). Chinese OP patients exhibit the enrichment of *Actinomyces* (e.g., *Actinomyces odontolyticus*) and *Streptococcus* (e.g., *Streptococcus sanguinis*), alongside the reduction of beneficial genera including *Akkermansia* and *Bacteroides*. Geographical dietary and lifestyle habits are likely the key contributing factors to these regional differences (5, 145).

Sex is also an important influencing factor. Female estrogen fluctuations act as a core regulatory factor: alterations in microbial diversity occur during pregnancy and lactation, and temporary bone loss accompanied by intestinal dysbiosis may increase the long-term risk of OP. Postmenopausal estrogen decline induces reduced microbial diversity and impaired intestinal barrier function, which exacerbates bone loss via the microbiota-gut barrier-immune axis (151). The incidence of OP is relatively low in males, yet decreased testosterone with aging leads to progressive BMD reduction, and males face higher rates of disability and mortality following fractures (152) (**Table 3**).

**Table 3:** Summary of clinical trials on changes in core gut microbiota in patients with osteoporosis.

Reference	Country/Region	Study type	Sample size	Detailed sample characteristics	Relative abundance changes of gut microbiota	
					(Increased)	(Decreased)
(146)	China	Cross-sectional Study	62	8 cases with normal bone mineral density (BMD); 23 cases with osteopenia; 31 cases with osteoporosis	Genus level: *Faecalibacterium*, *Agathobacter*, *Butyricimonas*, unclassified Glycomonadaceae bacteria, unclassified UCG–002 bacteria, unclassified *Ruminococcus*, *Subdoligranulum*; Species level: unclassified Faecalibacterium	*Prevotella copri*
(153)	China	Case-Control Study	63	25 patients with osteoporosis; 38 postmenopausal participants with normal BMD	Genus level: Agathobacter, *Lactobacillus*, Oscillibacter, Prevotellaceae_UGG-001	Genus level: *Bacteroides*, *Blautia*, *Fusicatenibacter*, Ruminococcus, *Anaerostipes*
(147)	Ireland	Case-Control Study	181	Osteopenia (n = 61); Osteoporosis (n = 60); Healthy (n = 60)	Genus level: *Actinomyces*, *Eggerthella*, *Clostridium* Cluster XlVa, *Lactobacillus*	-
(143)	China	Case-Control Study	108	44 cases of osteoporosis; 64 control cases	Phylum level: Bacteroidetes; Genus level: Clostridium Cluster XlVa, *Coprococcus*, *Lactobacillus*, Eggerthella, Bacteroides, *Eisenbergiella*	Genus level: *Veillonella*
(144)	China	Case-Control Study	15	5 healthy individuals; 10 patients with osteoporosis	Genus level: Clostridium	Genus level: *Lactobacillus*
(154)	China	Multistage Case-Control Study	180	77 patients with osteoporosis; 103 healthy controls	Phylum level: Bacteroidetes; Family level: Bacteroidaceae, Porphyromonadaceae; Genus level: Bacteroides, *Parabacteroides*	Phylum level: Firmicutes, Proteobacteria, Actinobacteria; Family level: Lachnospiraceae, Bifidobacteriaceae; Genus level: *Butyricicoccus*, *Bifidobacterium*
(155)	China	Case-Control Study	180	6 cases of primary osteoporosis; 6 cases of primary osteopenia; 6 normal controls	Phylum level: Significantly increased relative abundance of Firmicutes, Gemmatimonadetes, Chloroflexi; Genus level: Blautia, *Parabacteroides*, Ruminococcaceae genera (in primary osteoporosis group)	Phylum level: Bacteroidetes
(156)	South Korea	Case-Control Study	76	60 healthy controls; 16 patients with osteoporosis	Order level: Micrococcales; Family level: Micrococcaceae, Bacillaceae; Genus level: *Lachnospira*, *Planococcus*, PAC000195_g, PAC000741_g, PAC001435_g, PAC001231_g	-
(157)	China	Case-Control Study	58	21 postmenopausal patients with osteoporosis; 37 healthy controls	Phylum level: Fusobacteria; Class level: Relatively higher abundance of Bacilli, Erysipelotrichia; Order level: Lactobacillales; Family level: Lactobacillaceae; Genus level: *Lactobacillus*; Species level: *Lactobacillus salivarius*	Family level: Ruminococcaceae; Species level: Eggerthella lenta
(158)	China	Prospective Cohort Study	517	517 independent, unrelated perimenopausal/postmenopausal Chinese women	Genus level: *Bacteroides vulgatus* (relative abundance negatively correlated with lumbar spine L1-L4 BMD)	-
(159)	China	Case-Control Study	98	Non-postmenopausal osteoporosis subjects (n = 58); Newly diagnosed postmenopausal osteoporosis patients (n = 40)	Bacteria: Genus level: *Veillonella*, *Parabacteroides*, *Harryflintia*; Fungi: Genus level: *Eurotium*, *Penicillium*, *Chlorophyllum*	Bacteria: Genus level: Prevotella, Enterobacterium; Fungi: Genus level: *Pichia*, *Auricularia, Myrothecium*
(139)	China	Prospective Case-Control Study	96	48 cases of primary osteoporosis; 48 healthy controls	Phylum level: Bacteroidetes; Class level: Bacteroidia; Order level: Bacteroidales; Family level: Ruminococcaceae, Prevotellaceae; Genus level: Faecalibacterium, unidentified_Prevotellaceae, *Dialister*	Class level: Erysipelotrichia; Family level: Lachnospiraceae; Genus level: Subdoligranulum, Blautia, unidentified_Erysipelotrichaceae
(160)	Spain	Case-Control Observational Study	50	25 elderly patients with fragility hip fracture; 25 non-fracture patients	Phylum level: Bacteroidota; Order level: Bacteroidales, Peptostreptococcales-Tissierellales	Phylum level: Firmicutes; Order level: Oscillospirales, Lachnospirales
(145)	China	Case-Control Study	88	29 patients with osteoporosis; 59 participants with normal BMD	Species level: *Actinomyces gerencseriae*, *Actinomyces odontolyticus*, unclassified *Olsenella*, unclassified *Pantoea*, , *Streptococcus mitis/oralis/pneumoniae*, Streptococcus parasanguinis, *Streptococcus sanguinis*, pathogenic *Escherichia coli*	Species level: *Akkermansia muciniphila*, *Eggerthella lenta*, *Bacteroides fragilis*, *Bacteroides uniformis*, *Butyricimonas synergistica*

## 6 The gut-kidney-bone axis crosstalk: a gut microbiota-mediated regulatory network for kidney-bone interactions

### 6.1 Short-chain fatty acids – the “protective factors” of the gut-kidney-bone axis

SCFAs are produced by the gut microbiota through the fermentation of dietary fiber, primarily derived from the phyla Firmicutes and Bacteroidetes, and act as critical protective factors for maintaining the homeostasis of the gut-kidney-bone axis (161).

For the gut: butyrate, the main energy source for colonic epithelial cells, enhances the integrity of intestinal tight junction proteins (occludin, ZO-1), reduces intestinal permeability, inhibits the development of intestinal barrier leakage, and prevents the translocation of bacterial lipopolysaccharide (LPS) into the bloodstream (162). CKD patients exhibit a threefold reduction in serum butyrate levels compared with healthy individuals due to insufficient dietary fiber intake (caused by phosphorus and potassium dietary restrictions) and a decrease in SCFA-producing bacteria (e.g., *Faecalibacterium*, *Roseburia*), with serum butyrate levels negatively correlated with renal function (163). The above regulatory mechanisms implicated in anti-inflammatory and anti-fibrotic actions also exert critical protective effects on bone homeostasis and are closely associated with the maintenance of homeostasis in the gut-kidney-bone axis.

For the kidney: SCFAs inhibit the renal nuclear factor-κB (NF-κB) pathway by activating G protein-coupled receptors (GPR41, GPR43), reduce the release of pro-inflammatory cytokines such as interleukin-6 (IL-6) and tumor necrosis factor-α (TNF-α), and alleviate renal interstitial inflammation and fibrosis (163).

In terms of skeletal regulation, the modulatory effects of SCFAs on host biological responses are primarily mediated through two molecular pathways. The primary mechanism involves the inhibition of histone deacetylases (HDACs), which achieves epigenetic regulation by blocking HDAC-mediated histone modification processes (164). The second pathway entails G protein-coupled receptor (GPCR)-mediated signal transduction, which not only drives the differentiation of bone marrow naive CD4+ T cells into T regulatory cells (Tregs) but also synergizes with the transforming growth factor-β (TGF-β) signaling cascade to simultaneously activate nuclear factor of activated T cells (NFAT) and SMAD signaling molecules (164).

These transcription factors specifically bind to the promoter region of the Wnt10b gene, significantly enhancing its transcriptional activity and ultimately inducing the lineage-specific differentiation of osteoblasts. Microbe-derived butyrate, as a key effector molecule, precisely regulates Wnt10b production via the TGF-β signaling axis by mediating the interaction network between Tregs and bone marrow CD8+ T cells, thereby enhancing osteogenesis in juvenile animals (165).

In recent studies using rat models, Chen X et al. found that lactulose inhibited osteoclastogenesis and bone resorption by altering the gut microbiota and increasing SCFA levels (166).

Zhang Z et al. verified that administration of fructooligosaccharides (FOS) and/or galactooligosaccharides (GOS) significantly increased the microbial diversity and SCFA concentrations in the gut microbiota of high-fat diet (HFD)-fed mice, which in turn reversed intestinal hyperpermeability and elevated inflammatory cytokines, ultimately preventing HFD-induced osteopenia (167). Lucas S et al. demonstrated that direct SCFA treatment in mice for 8 weeks resulted in a marked increase in bone mass and a significant reduction in bone-resorbing osteoclasts (168). Other studies have shown that inulin supplementation promotes active fermentation in the large intestine and the proliferation of lactic acid bacteria, as well as enhancing the digestion, absorption, and deposition of calcium in bone, which is beneficial for osteogenesis (169).

In summary, SCFAs play a pivotal role in skeletal health. However, the scientific community remains divided on the causes of reduced SCFA levels: several studies have indicated that CKD impairs the capacity for SCFA production (44, 170), while others propose that this reduction may be attributed to low dietary fiber intake rather than a diminished ability to produce SCFAs (171, 172).

### 6.2 Uremic toxins – the “destructive factors” of the gut-kidney-bone axis

In CKD, uremic toxins generated by the gut microbiota through amino acid metabolism accumulate due to impaired renal excretion, emerging as core mediators that exacerbate renal injury and bone metabolic abnormalities.

In terms of renal injury: serum IS concentrations can increase 10- to 100-fold in CKD patients (25). IS induces oxidative stress and activates the NF-κB pathway in renal cells, damages renal tubular epithelial cells and renal endothelial cells, and accelerates renal fibrosis (173). pCS promotes monocyte-endothelial cell interactions and exacerbates renal vascular inflammation (174).

The effects on bone metabolism are multifaceted: IS directly inhibits osteoblast differentiation and bone mineralization, and reduces alkaline phosphatase (ALP) activity and the expression of osteogenesis-related genes (e.g., Runx2, OCN) (28, 175). It also suppresses osteoclast function, leading to low-turnover bone disease characterized by reduced both bone formation and bone resorption (176, 177). Watanabe K et al. reported that IS exacerbates parathyroidectomized (PTX)-induced low bone turnover in adult rats and proposed that IS directly induces low bone turnover by inhibiting osteogenesis, a mechanism independent of skeletal resistance to PTH. IS may directly act on osteoblasts and osteoclast precursor cells to suppress both bone formation and resorption, thereby contributing to the development of low-turnover bone disease in CKD patients (178).

During osteogenic differentiation, high concentrations of uremic toxins lead to downregulated type I collagen expression, diminished ALP activity, and impaired mineralization capacity in mesenchymal stem cells (MSCs) (179), which corroborates the adverse effects of uremic toxins on osteogenesis. Additionally, elevated IS concentrations activate 24-hydroxylase (CYP24A1), accelerating the degradation of 25-hydroxyvitamin D and active vitamin D, and resulting in decreased calcitriol (1,25-dihydroxycholecalciferol) levels (180).

In the choline metabolic pathway, specific gut microbiota such as the phylum Proteobacteria convert dietary choline, phosphatidylcholine, and L-carnitine into trimethylamine (TMA). This gaseous molecule is absorbed via the portal system and catalyzed by hepatic flavin-containing monooxygenase 3 (FMO3) to form the final product TMAO (181). TMAO disrupts endoplasmic reticulum autophagy by activating the protein kinase R-like endoplasmic reticulum kinase (PERK)/activating transcription factor 4 (ATF4)-dependent signaling pathway and inhibits the ATF5-mediated mitochondrial unfolded protein response, thereby impairing osteoblast differentiation and mineralized matrix synthesis capacity, and ultimately exacerbating bone loss (182).

### 6.3 Vitamin K – the “mineralization modulator” of the gut-kidney-bone axis

As a fat-soluble vitamin, vitamin K acts as a coenzyme for γ-glutamyl carboxylase (GGCX) to activate vitamin K-dependent proteins including osteocalcin and matrix Gla protein (MGP), thereby playing a pivotal role in the regulation of bone metabolism and maintenance of mineralization homeostasis (183). Clinical studies have confirmed that subclinical vitamin K deficiency is prevalent in patients with chronic kidney disease (CKD), which is mainly characterized by elevated serum levels of undercarboxylated osteocalcin (%ucOC) and protein induced by vitamin K absence or antagonist-II (PIVKA-II) (184). The core contributing factors include dietary restrictions (insufficient intake of leafy vegetables due to phosphorus and potassium control), drug interference (antibiotics, phosphate binders, etc.), and reduced endogenous synthesis caused by intestinal microbial dysbiosis (185).

Vitamin K exists in two major bioactive forms: plant-derived vitamin K1 (phylloquinone, PK) and microbe-derived vitamin K2 (menaquinones, MKs). It regulates bone metabolism and mineralization homeostasis through both γ-carboxylation-dependent and -independent mechanisms. On the one hand, as a coenzyme for GGCX, it specifically catalyzes the γ-carboxylation modification of osteocalcin (OC) and MGP—carboxylated OC can specifically bind to hydroxyapatite to promote bone matrix mineralization, while carboxylated MGP effectively inhibits ectopic calcification of blood vessels and other tissues, indirectly maintaining bone metabolic homeostasis (183, 186, 187). On the other hand, vitamin K2 (MK-4) acts as a nuclear receptor ligand to activate the steroid and xenobiotic receptor (SXR), referred to as the pregnane X receptor (PXR) in rodents, thereby inducing the expression of osteogenesis-related genes such as Matn2 and Tsk in osteoblasts and maintaining the material mechanical properties of bone by regulating collagen cross-linking. Meanwhile, it inhibits osteoclast differentiation by antagonizing the NF-κB signaling pathway, achieving bidirectional regulation of bone metabolism (188, 189).

As early as 1997, studies explored the association between poor vitamin K status and skeletal health in CKD patients. A survey of 68 hemodialysis patients by Kohlmeier M et al. showed that vitamin K nutritional status was closely correlated with biochemical indicators of bone metabolism, and vitamin K deficiency (characterized by decreased phylloquinone levels and reduced carboxylation rate of serum osteocalcin) had an independent and strong correlation with a high risk of fractures (190). Another observational study of 387 hemodialysis patients further confirmed that the vitamin K system is of great significance for maintaining bone mass and preventing vascular calcification in this population. The researchers suggested screening for vitamin K deficiency in the general population, as it may be a potential risk factor for vertebral fractures and vascular calcification (191).

Vitamin K deficiency is highly prevalent in CKD patients, with core causes including insufficient exogenous intake due to dietary restrictions (reduced consumption of leafy vegetables for potassium and phosphorus control), interference from long-term use of drugs such as antibiotics, phosphate binders, iron supplements and immunosuppressants, and further reduced endogenous synthesis aggravated by altered intestinal microbiota composition. These factors ultimately lead to elevated levels of undercarboxylated osteocalcin (unOC) and increased bone fragility (185, 192).

In mechanistic studies on how altered intestinal microbiota composition exacerbates vitamin K deficiency, a Japanese research team investigated the association between intestinal microbiota and a surrogate marker of vitamin K deficiency (serum undercarboxylated osteocalcin) in patients with clinically quiescent Crohn’s disease. They found that patients with vitamin K deficiency had a significant reduction in intestinal microbial alpha diversity, along with markedly decreased abundances of Ruminococcaceae and Lachnospiraceae (193). Animal experiments by Guss JD et al. further confirmed that male C57Bl/6 mice with disrupted intestinal microbiota had a 32% to 66% reduction in the levels of microbe-derived vitamin K (menaquinones, MKs) in the cecum, liver and kidney compared with untreated mice (194). In addition, intestinal microbial dysbiosis has also been observed in patients with osteoporosis, and the levels of biomarkers associated with vitamin K deficiency are significantly elevated in this population (195). The above studies collectively indicate that intestinal microbial homeostasis is crucial for maintaining host vitamin K status.

### 6.4 Vitamin D – the “core regulator of calcium and phosphate metabolism” in the gut-kidney-bone axis

As a steroid derivative, vitamin D is a fat-soluble essential nutrient with hormonal activity, playing a central role in the regulation of calcium and phosphate metabolism, maintenance of skeletal health, and various physiological processes in the human body. The human body obtains vitamin D mainly through two pathways: *de novo* biosynthesis in the skin upon exposure to ultraviolet B (UVB) radiation, and dietary supplementation. When the skin is exposed to UVB, 7-dehydrocholesterol in the epidermis is converted to previtamin D3 via photochemical reaction, which is then transformed into vitamin D3 (cholecalciferol) through non-enzymatic thermal isomerization (196). After entering the blood circulation, vitamin D3 forms a complex with vitamin D-binding protein (DBP) and is transported to the liver via the portal system. It undergoes the first hydroxylation catalyzed by 25-hydroxylase (mainly CYP27A1) in the microsomes of hepatocytes to generate 25-hydroxyvitamin D3 (25(OH)D3)—the major circulating form of vitamin D, which remains bound to DBP and is further transported to the kidney through the blood circulation (197). In the epithelial cells of the renal proximal convoluted tubule, the second hydroxylation is catalyzed by 1α-hydroxylase (CYP27B1) to produce 1,25-dihydroxyvitamin D3 (1,25(OH)_2_D3), the fully bioactive form of vitamin D (198).

Vitamin D deficiency plays a key role in mineral and bone metabolic disorders in CKD patients (197) Studies have confirmed that vitamin D deficiency is common in CKD patients even in the early stages of the disease, and this deficiency gradually worsens with the progression of renal function (199). In pediatric CKD patients, vitamin D deficiency easily induces secondary hyperparathyroidism (SHPT), which disrupts bone metabolic homeostasis by accelerating bone turnover (200). A study by Denburg MR et al. on the influencing factors of cortical bone volumetric bone mineral density (CortBMD) in pediatric CKD patients further confirmed that higher levels of parathyroid hormone (PTH) and 1,25(OH)_2_D3, as well as lower calcium concentrations, were independently associated with reduced baseline CortBMD and progressive cortical bone damage in these patients (200).

1,25(OH)_2_D3, the active form of vitamin D, directly participates in osteocyte differentiation and bone matrix mineralization by regulating calcium and phosphate balance and PTH levels, and is thus critical for maintaining bone metabolic homeostasis. To investigate the effects of oral cholecalciferol (vitamin D3) supplementation on mineral metabolism and bone turnover markers in a specific population, Yadav AK et al. conducted a randomized, double-blind, placebo-controlled trial involving 120 non-diabetic CKD patients at stages G3-G4. The final results showed that cholecalciferol supplementation effectively corrected vitamin D deficiency, not only inhibiting SHPT but also significantly improving biochemical indicators related to mineral metabolism, while reducing the levels of bone turnover markers (serum total alkaline phosphatase, bone-specific alkaline phosphatase, and C-telopeptide of type I collagen) (201).

There is a close bidirectional regulatory relationship between vitamin D and the intestinal microbiota. Studies have shown that probiotics can not only directly enhance the vitamin D receptor (VDR) signaling pathway to inhibit intestinal inflammation but also effectively increase host vitamin D levels. A study by Wu S et al. found that *Lactobacillus rhamnosus GG* (LGG) and *Lactobacillus plantarum* (LP) not only upregulated VDR protein expression, enhanced VDR transcriptional activity and the transcription levels of target genes in intestinal epithelial cells of mice and humans but also increased the number of Paneth cells that secrete antimicrobial peptides and participate in host defense. Ultimately, they enhanced the resistance of mice to *Salmonella*-induced colitis through a VDR-dependent pathway (202). Another randomized, double-blind, placebo-controlled trial by Jones ML et al. first reported that oral supplementation with *Lactobacillus reuteri* NCIMB 30242 in healthy adults with hypercholesterolemia resulted in a significant 25.5% increase in serum 25-hydroxyvitamin D (25(OH)D) levels, an effect independent of the placebo group (203). Conversely, vitamin D deficiency can induce intestinal microbial dysbiosis, characterized by decreased abundances of beneficial bacteria such as *Lactobacillus* and *Bifidobacterium* (204–206). However, existing research data on the effects of dietary vitamin D supplementation on the intestinal microbiota are clearly contradictory: a randomized, double-blind, placebo-controlled trial in elderly individuals aged 60–84 years showed that monthly supplementation with 60,000 IU of vitamin D3 for 5 years did not significantly alter the overall composition or alpha/beta diversity of the intestinal microbiota (207). In contrast, a study on overweight/obese adults with vitamin D deficiency (25(OH)D ≤ 50 nmol/L) found that cholecalciferol supplementation (a loading dose of 100,000 IU plus 4,000 IU daily for 16 weeks) led to increased abundance of *Lachnospira* and decreased abundance of *Blautia* in the fecal microbiota. Additionally, the abundance of *Coprococcus* was significantly higher in individuals with serum 25(OH)D > 75 nmol/L than in those with 25(OH)D < 50 nmol/L (208).

Overall, the heterogeneity of study results may be related to key variables such as age stratification and gender composition of the study populations. Therefore, a unified conclusion has not yet been reached regarding the specific effects of dietary vitamin D supplementation on the intestinal microbial community structure, and more targeted studies are needed to further clarify this issue.

### 6.5 Bile acids: core signaling molecules mediating gut-kidney-bone axis crosstalk via the gut microbiota

Bile acids (BAs), core cholesterol metabolites, act as key hubs for gut microbiota-mediated crosstalk in the gut-kidney-bone axis (209, 210). Liver-synthesized primary BAs—mainly cholic acid and chenodeoxycholic acid, mostly glycine- or taurine-conjugated—enter the intestine. Approximately 95% are reabsorbed via active ileal transport to complete enterohepatic circulation, while the remaining 5% serve as microbial substrates. Catalyzed by bile salt hydrolase (BSH) and 7α-dehydroxylase, these primary BAs undergo deconjugation, dehydroxylation, and epimerization to form secondary BAs (e.g., deoxycholic acid [DCA], lithocholic acid [LCA], ursodeoxycholic acid [UDCA]), composing a dynamically balanced BA pool that sustains gut-liver-microbiota homeostasis (54, 211). By specifically binding and activating nuclear receptors (farnesoid X receptor [FXR], vitamin D receptor [VDR]) and membrane receptor G protein-coupled receptor TGR5, BAs mediate downstream signaling to enable gut-kidney-bone cross-talk, maintaining metabolic and immune homeostasis (212–216).

For the intestine, secondary BAs (e.g., isoDCA, 3-oxoLCA, isoalloLCA) maintain homeostasis through dual mechanisms: on the one hand, they preserve the integrity of tight junctions via the FXR/TGR5 pathway in intestinal epithelial cells, reducing intestinal leakage and ectopic translocation of lipopolysaccharide (LPS) (217); on the other hand, they attenuate the immunostimulatory activity of dendritic cells (DCs) via the FXR pathway, or directly bind to the transcription factor RORγt of Th17 cells and mediate mitochondrial reactive oxygen species (ROS) production in Treg cells, precisely regulating the Treg/Th17 balance to maintain intestinal mucosal immune homeostasis (217–219).

For the kidney, kidney-specific FXR activation inhibits the progression of renal interstitial fibrosis and the release of pro-inflammatory cytokines such as TNF-α and IL-6 by mediating downstream anti-inflammatory and anti-fibrotic signaling pathways, thus maintaining renal metabolic homeostasis. In pathological states such as intestinal microbial dysbiosis, excessive accumulation of BAs including LCA and taurochenodeoxycholic acid (TCDCA) blocks this protective effect by competitively antagonizing renal FXR activity, thereby exacerbating renal tissue damage (220–222).

For the skeleton, UDCA and chenodeoxycholic acid (CDCA) can upregulate the expression of osteogenic markers such as RUNX2 and osteocalcin (OC) by activating the FXR pathway in osteoblasts and osteocytes, or promote osteoblast proliferation and inhibit osteoclast differentiation via the TGR5-mediated cAMP-PKA pathway. Meanwhile, UDCA can neutralize the bone-damaging effects of LCA and other BAs, restoring osteocyte viability and mineralization function. In contrast, LCA impairs the differentiation of osteoblasts and osteocytes and the process of bone mineralization by interfering with mitochondrial function, upregulating RANKL expression (to promote osteoclast activation), and competitively binding to VDR to antagonize its mediated signaling pathway (223, 224).

During intestinal microbial dysbiosis, the imbalance in the composition of the bile acid pool disrupts the homeostatic regulation of the gut-kidney-bone axis, serving as an important mediating mechanism for the comorbidity of diseases such as CKD, osteoporosis (OP) and osteoarthritis. This highlights the core mediating role of BAs in the microbiota-organ interaction network (220, 225, 226).

### 6.6 Intestinal mucosal barrier – the “key hub” for gut-kidney-bone axis dysregulation

Intestinal microbial dysbiosis can disrupt the integrity of the intestinal mucosal barrier, triggering a cascade of pathological reactions of “intestinal leakage - endotoxemia - systemic inflammation”, which acts as a core conduction pathway between intestinal injury and renal-bone damage. The structural homeostasis of the intestinal mucosal barrier is mainly maintained by tight junction proteins between intestinal epithelial cells (ZO-1, occludin, and the claudin family). Downregulated expression of these proteins directly leads to increased intestinal permeability, creating conditions for the invasion of bacteria and endotoxins (227).

Under dysbiotic conditions, pathogenic bacteria such as *Salmonella* and *Escherichia coli* proliferate extensively, releasing endotoxins such as LPS and metabolic waste products, while inducing host monocytes/macrophages to produce pro-inflammatory cytokines such as TNF-α and IL-6, which further exacerbate intestinal barrier damage (228). Animal experiments have confirmed that the intestinal microbiota and its products (e.g., peptidoglycan, LPS) positively regulate the integrity of the mucus barrier: the colonic mucus layer is significantly thinner in germ-free mice, and supplementation with bacterial products can restore its structure (14). Conversely, studies using fecal microbiota transplantation (FMT) rat models by Zhang YW and Ma S both observed that the expression of intestinal epithelial tight junction proteins (occludin, claudin and ZO-1) was significantly reduced in the osteoporotic state, and correction of microbial dysbiosis via FMT not only improved intestinal barrier function but also alleviated bone loss (229, 230).

Systemic inflammation is the key link connecting intestinal barrier damage and renal-bone abnormalities: a study by Kobayashi K found that elevated TNF-α levels can stimulate osteoclastogenesis and/or promote osteoblast apoptosis (231). Itoh K et al. further confirmed that in the presence of macrophage colony-stimulating factor (M-CSF), TNF-α can directly stimulate osteoclast differentiation through a mechanism independent of the osteoclast differentiation factor (ODF, also known as RANKL)-RANK signaling system (232). A clinical study by Viaene L et al. provided evidence for the notion that “inflammation is the core link of gut-kidney-bone axis dysregulation”—in patients with end-stage renal disease (ESRD), serum IL-6 levels were independently correlated with aortic calcification, and TNF-α levels were independently associated with low bone area (233). In addition, mechanistic studies by Hou GQ et al. confirmed that LPS can dualistically promote osteoclast differentiation and functional activation by enhancing RANK signal transduction and COX-2 expression, while activating the JNK and ERK1/2 MAPK signaling pathways (234).

In summary, the regulation of intestinal permeability is a key link in the maintenance of gut-kidney-bone axis homeostasis. The intestinal microbiota indirectly participates in the regulation of host bone metabolism and renal function through inflammatory pathways by modulating the integrity of the mucosal barrier, and its imbalance is an important pathological basis for the development and progression of CKD-MBD.

### 6.7 Endocrine factor-mediated regulation – bidirectional feedback of the FGF23-Klotho axis and PTH

The intestinal microbiota participates in the long-distance regulation of the gut-kidney-bone axis by modulating endocrine factors such as fibroblast growth factor 23 (FGF23), Klotho protein and parathyroid hormone (PTH), and the imbalance of these factors is a core feature of chronic kidney disease-mineral and bone disorder (CKD-MBD) (235).

The FGF23-Klotho axis is a core endocrine regulatory pathway for maintaining phosphorus metabolic balance and bone homeostasis, and also a key node for the intestinal microbiota to participate in the homeostatic regulation of the gut-kidney-bone axis (236, 237). Under physiological conditions, FGF23 secreted by osteoblasts/osteocytes must form a complex with Klotho protein (a co-receptor) and FGFR1 on the surface of renal distal convoluted tubule epithelial cells to specifically inhibit the expression and function of sodium-phosphate cotransporters (NaPi-2a/2c), reduce renal phosphorus reabsorption and maintain circulating phosphorus homeostasis. Meanwhile, soluble Klotho (sKlotho) secreted by the kidney can reversely stimulate bone tissue to synthesize FGF23, forming a bone-kidney bidirectional feedback loop that synergistically regulates vitamin D activation and bone metabolic balance (235, 238).

In the CKD state, the homeostasis of this axis is disrupted by intestinal microbial dysbiosis: uremic toxins such as trimethylamine N-oxide (TMAO) and indoxyl sulfate (IS) produced by intestinal microbial metabolism accumulate and directly inhibit renal Klotho protein expression, resulting in the failure of FGF23 to bind to its receptor effectively, a state termed “FGF23 resistance” (235). To compensate for impaired phosphorus excretion, bone tissue secretes excessive FGF23, and persistently elevated FGF23 further inhibits renal 1α-hydroxylase activity, reduces the synthesis of active vitamin D (1,25(OH)_2_D3), and exacerbates calcium and phosphorus metabolic disorders and bone remodeling imbalance. At the same time, sKlotho levels are significantly decreased in CKD, leading to the disruption of the bone-kidney feedback loop, which ultimately amplifies multi-organ damage of the gut-kidney-bone axis and acts as a core driving factor for the progression of CKD-MBD (239, 240).

Parathyroid hormone (PTH) is a core hormone regulating calcium and phosphorus metabolism and skeletal homeostasis: it maintains blood calcium balance by promoting renal calcium reabsorption, enhancing bone resorption to release calcium, and activating the vitamin D pathway to facilitate intestinal calcium absorption (241). Meanwhile, PTH exerts bidirectional regulatory effects on bone metabolism—persistent high expression leads to bone loss, while intermittent PTH (iPTH) administration is an FDA-approved bone anabolic therapy that can effectively promote bone formation (242). Notably, the osteogenic effect of iPTH is dependent on the participation of the intestinal microbiota, a finding that has become a research focus in the field of the gut-bone axis in recent years. The intestinal microbiota produces short-chain fatty acids (SCFAs) through the fermentation of dietary fiber, which provides an essential condition for iPTH to exert its bone-regulatory effects (243). Butyrate enhances antigen presentation efficiency by activating the GPR43 receptor on the surface of dendritic cells and directly induces the differentiation of CD4+ T cells into regulatory T cells (Tregs) (242). These Tregs further stimulate bone marrow CD8+ T cells to secrete Wnt10b, a key osteogenic signaling molecule, which promotes the proliferation and differentiation of osteoprogenitor cells and the deposition of mineralized matrix by activating the canonical Wnt/β-catenin pathway (243). Animal experiments have confirmed that in germ-free mice or mice with antibiotic-induced microbiota depletion, circulating butyrate levels are significantly reduced, and iPTH fails to induce increased osteoblast activity and bone formation; restoration of physiological butyrate levels can fully reconstitute the osteogenic effect of iPTH (244, 245). A study by the Li JY team further verified this mechanism and identified butyrate as the key mediator linking the intestinal microbiota and the osteogenic effect of iPTH (243).

Interestingly, the secretory pattern of PTH exerts a significant bidirectional regulatory effect on the intestinal microbiota: intermittent PTH (iPTH, physiological pulsatile secretion) can reshape the intestinal microbial structure to form a positive regulatory loop of “iPTH - beneficial microbiota - butyrate - bone formation”. Rat experiments by Zhou J et al. confirmed that iPTH treatment significantly increased the abundances of known bone mass-promoting microbiota including *Lactobacillus reuteri*, Muribaculaceae, Ruminococcaceae and Clostridia, while inhibiting the relative abundance of Rikenellaceae, which is potentially associated with osteoporosis. Tax4Fun functional prediction further showed that iPTH could specifically upregulate the butyrate synthesis metabolic pathway of the microbiota (246).

In contrast, pathological persistent high PTH exposure (e.g., primary hyperparathyroidism) induces intestinal microbial dysbiosis. A study by Yu M et al. found that under the action of persistent PTH, the enrichment of segmented filamentous bacteria (SFB) promotes the expansion of intestinal TNF^+^ T cells and Th17 cells, which migrate to the bone marrow and activate osteoclasts by secreting pro-inflammatory cytokines, disrupting bone metabolic balance (247). This pathological cascade is microbiota-dependent: persistent PTH fails to induce bone loss in germ-free mice, and fecal microbiota transplantation (FMT) can reconstitute this bone metabolic response, directly confirming the existence and functional relevance of the microbiota-PTH-bone axis.

**Figure 4:**
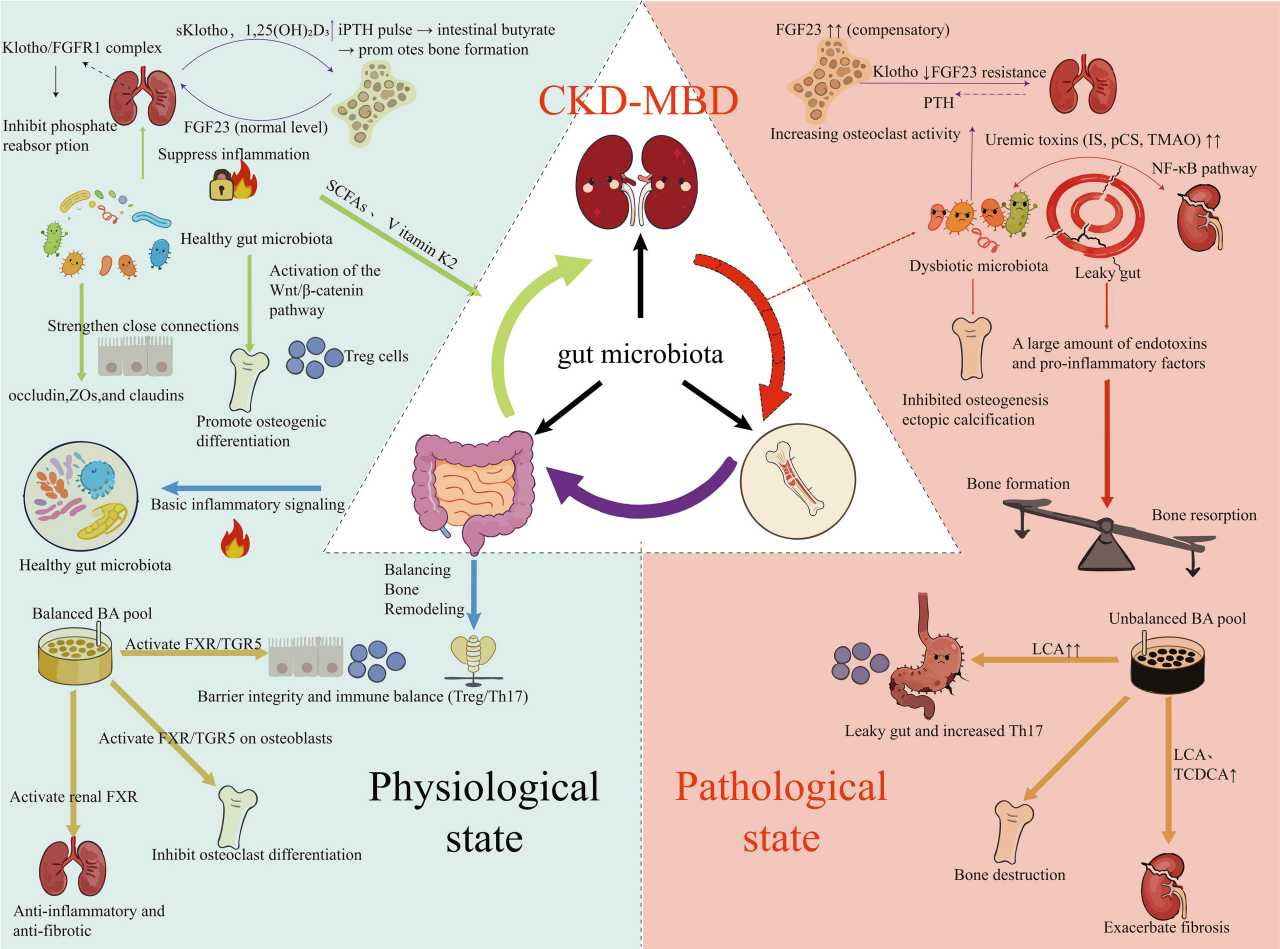
Schematic diagram of gut microbiota-mediated bidirectional gut-kidney-bone crosstalk in physiological homeostasis and the pathogenesis of chronic kidney disease-mineral and bone disorder (CKD-MBD). This triangular model illustrates the dynamic interactions of the gut-kidney-bone axis, highlighting the central role of the gut microbiota in regulating renal and bone metabolism by comparing physiological and pathological states. In the physiological state, a healthy gut microbiota maintains intestinal barrier integrity via tight junction proteins (occludin, ZO proteins, and claudins), activates the Wnt/β-catenin pathway to promote osteogenic differentiation, and sustains immune homeostasis through regulatory T cells (Tregs). Short-chain fatty acids (SCFAs) and vitamin K2 further enhance bone formation; meanwhile, a balanced bile acid (BA) pool activates the FXR/TGR5 signaling pathway, inhibits osteoclast differentiation, and exerts anti-inflammatory and anti-fibrotic effects on the kidneys. The kidneys suppress phosphate reabsorption and reduce inflammation via the Klotho/FGFR1 complex, while participating in maintaining bone homeostasis through physiological levels of fibroblast growth factor 23 (FGF23).In the pathological state (light red area), dysbiotic microbiota induces intestinal barrier dysfunction (“leaky gut”), releasing uremic toxins (indoxyl sulfate [IS], p-cresyl sulfate [pCS], trimethylamine N-oxide [TMAO]) and pro-inflammatory factors. This activates the NF-κB pathway, leading to a shift in bone metabolism toward excessive bone resorption, impaired bone formation, and ectopic calcification. Compensatory elevation of FGF23 combined with Klotho deficiency causes FGF23 resistance, while increased parathyroid hormone (PTH) levels enhance osteoclast activity. An imbalanced bile acid pool (elevated levels of lithocholic acid [LCA] and taurochenodeoxycholic acid [TCDCA]) exacerbates leaky gut, bone destruction, and renal fibrosis, forming a pathological feedback loop that drives CKD-MBD progression. The central triangular framework emphasizes the gut microbiota as a key regulator linking renal and bone metabolism, with bidirectional arrows representing interactions that either maintain homeostasis or drive disease progression. CKD-MBD, Chronic Kidney Disease-Mineral and Bone Disorder; FGFR1, Fibroblast Growth Factor Receptor 1; 1,25(OH)_2_D_3_, 1,25-dihydroxyvitamin D_3_; iPTH, intact Parathyroid Hormone; SCFAs, Short-Chain Fatty Acids; Treg, Regulatory T Cell; BA, Bile Acid; FXR, Farnesoid X Receptor; TGR5, G Protein-Coupled Bile Acid Receptor 5; Th17, T Helper 17 Cell; FGF23, Fibroblast Growth Factor 23; IS, Indoxyl Sulfate; pCS, p-Cresyl Sulfate; TMAO, Trimethylamine N-Oxide; NF-κ B, Nuclear Factor kappa-B; LCA, Lithocholic Acid; TCDCA, Taurochenodeoxycholic Acid.

In summary, the discovery that the intestinal microbiome mediates the effects of PTH on the skeleton provides new insights for the treatment of metabolic bone diseases. Targeting the gut microbiota-butyrate axis may enhance the bone-forming effect of PTH analogs and simultaneously inhibit bone loss caused by long-term PTH exposure. Current studies have confirmed that supplementation with butyrate precursors or specific probiotics can significantly improve bone microstructural parameters (bone mineral density, bone volume fraction, etc.) in osteoporotic model animals, and these effects show promising translational medical potential (244, 248) (**Figure 4**). 

## 7 Shared and disease-specific characteristics of the core gut microbiota across chronic diseases

Although chronic kidney disease (CKD), rheumatoid arthritis (RA), osteoarthritis (OA), and osteoporosis (OP) differ in their pathological mechanisms and target organs, an in-depth analysis of their intestinal microbial dysbiosis characteristics allows the identification of shared dysbiosis modules and disease-specific microbial signatures (3). This coexistence of shared and disease-specific features reveals the dual role of the gut microbiota as both a “systemic amplifier” and a “disease director” in systemic disorders.

### 7.1 Shared dysbiosis modules: the microbial basis of chronic inflammation and metabolic dysregulation

Intestinal microbial dysbiosis across these diseases shares the following core features, which collectively contribute to a systemic pathological background. First, there is an expansion of pro-inflammatory, endotoxin-rich microbiota. The phylum Proteobacteria, particularly members of the Enterobacteriaceae family, exhibits an enrichment trend in patients with CKD, RA, OA, and subsets of OP populations (39, 40, 79, 93, 121, 141). Bacteria of this phylum are the primary source of lipopolysaccharide (LPS), and their expansion directly exacerbates endotoxemia. By activating the systemic Toll-like receptor (TLR) signaling pathway, they continuously drive low-grade chronic inflammation—a core shared pathway linking intestinal, renal, bone, and joint injury (12, 21, 228). Second, these diseases are universally accompanied by the impairment of intestinal beneficial metabolic functions. Core commensal genera that produce short-chain fatty acids (SCFAs) are generally depleted: *Roseburia* and *Faecalibacterium* are significantly reduced in CKD and OP (39, 50, 146), while members of the Lachnospiraceae family associated with butyrate production also show a declining trend in RA and CKD (39, 48, 110). Insufficient SCFA synthesis directly impairs their roles in maintaining intestinal barrier integrity, promoting systemic anti-inflammatory immunity, and directly regulating bone metabolism, leading to attenuated protective signaling across multiple organs (2, 161, 164). Ultimately, the aforementioned dual imbalance of microbiota and metabolism collectively leads to the disruption of intestinal barrier integrity. Altered microbial composition and the consequent imbalance in metabolites cause the downregulated expression of tight junction proteins (e.g., occludin, ZO-1), exacerbating intestinal leakage (14, 227). Increased intestinal permeability serves as the physical basis for the ectopic translocation of bacterial products and antigens, providing a common pathological starting point for the activation of autoimmunity and inflammation in distal organs (kidney, joints, bone) (13, 16, 228).

### 7.2 Disease-specific microbial characteristics

Against the backdrop of shared dysregulation, abnormal changes in specific genera are closely coupled with the core pathological processes of each disease, forming disease-specific “microbial fingerprints”.

In CKD, the specific feature of microbial dysbiosis is the targeted enrichment of microbiota that synthesize uremic toxins. The increased abundance of *Bacteroides* is directly associated with the production of precursors of IS and p-CS (53, 56); the enrichment of *Akkermansia* in advanced CKD is positively correlated with renal function indices (51, 55). These changes, together with the loss of renal excretory function, form the unique pathological loop of “gut-derived uremic toxin accumulation”, which directly drives renal injury and CKD-mineral and bone disorder (CKD-MBD) (21, 173). For RA, the most characteristic microbial changes focus on autoantigen mimicry and impaired immune tolerance. *Prevotella* is significantly enriched in preclinical and early RA patients and is associated with the production of anti-cyclic citrullinated peptide antibodies (ACPA) (70, 71, 89, 97). The enrichment of *Collinsella* is closely linked to the generation of citrullinated antigens and the enhancement of intestinal Th17 immune responses (81, 82, 100). These microbial features directly participate in the initiation and amplification of autoimmune responses in RA (4, 83). Microbial dysbiosis in OA is more focused on driving local and systemic low-grade inflammation. Its specific manifestations include the enrichment of potential pathogenic bacteria such as *Escherichia coli*, *Klebsiella pneumoniae*, and *Streptococcus salivarius* (121, 123), accompanied by a reduction in the specific beneficial bacterium *Bacteroides* vulgatus (121, 130). This dysbiosis pattern may indirectly exacerbate the degeneration of articular cartilage and synovial inflammation by increasing the systemic inflammatory burden, rather than triggering typical autoimmune responses (115, 127, 128). In OP, the core of microbial changes is associated with the regulation of systemic bone metabolic homeostasis. The characteristic features include a decreased Firmicutes/Bacteroidetes (F/B) ratio (5, 140, 154) and the enrichment of *Eggerthella* and *Clostridium_XlVa* (143, 147). These changes may specifically affect the dynamic balance of osteoblasts and osteoclasts by interfering with bile acid circulation, influencing the metabolism of vitamins K and D, and regulating sex hormone levels, leading to impaired bone formation or excessive bone resorption (143, 145, 212, 213). In summary, the gut microbiota acts as a “shared disruptor” in CKD, RA, OA, and OP, creating a systemic environment for disease development through pro-inflammatory and metabolic dysregulation modules; it also serves as a “disease-specific driver”, directing systemic dysregulation toward organ-specific pathology through unique microbiota-host interaction patterns. Identifying these shared and disease-specific signatures is of great significance for developing anti-inflammatory microecological modulatory strategies and precision microbiota-targeted therapies for individual diseases.

## 8 Interventional strategies

Currently, intervention strategies mainly cover four aspects: live bacteria supplementation, dietary substrate regulation, application of microbial metabolites, and overall microbiota transplantation.

In terms of live microorganism supplementation, probiotics exert bone-protective effects through competitive colonization, enhancing intestinal barrier integrity, and regulating systemic immunity. In sex hormone deficiency models, *Lactobacillus rhamnosus* GG (LGG) and the composite probiotic preparation VSL#3 completely blocked estrogen deficiency-induced bone loss by reducing intestinal permeability and inhibiting inflammatory pathways (245). Notably, there is host specificity in strain effects: studies have found that *Lactobacillus reuteri* ATCC PTA 6475, which has anti-TNF-α activity, can significantly increase the bone formation rate and trabecular bone volume in healthy male mice, while no equivalent effect was observed in female mice (249). This concept has been successfully translated into clinical practice: a randomized controlled trial in early postmenopausal women showed that 12-month supplementation with a composite probiotic containing *Lactobacillus* paracasei and *Lactobacillus plantarum* effectively slowed lumbar spine bone mineral density (BMD) loss with good safety (250).

Prebiotics and postbiotics act on bone indirectly or directly by regulating microbial metabolic functions. Prebiotics (e.g., inulin, fructooligosaccharides [FOS]) are fermented by intestinal commensal bacteria to produce SCFAs, which in turn regulate bone metabolism. In ovariectomized rats, supplementation with a mixture of inulin and FOS not only significantly promoted calcium absorption and retention but also simultaneously increased femoral calcium content, BMD, and improved bone balance (251). Clinical studies have further shown that supplementation with FOS combined with calcium in postmenopausal women can more significantly reduce bone resorption markers; in the osteopenic subgroup, this regimen also effectively slowed spinal and total body BMD loss (252). More directly, supplementation with propionate—a representative postbiotic of SCFAs—significantly increased the bone formation marker osteocalcin and decreased the bone resorption marker β-crosslaps (β CTx) in patients with multiple sclerosis, highlighting the potential of microbial metabolites to directly regulate bone homeostasis (253).

Dietary therapy represents a core strategy for modulating the gut microbiota in patients with chronic kidney disease-mineral and bone disorder (CKD-MBD). Owing to potassium- and phosphorus-restricted diets, patients with CKD often exhibit insufficient dietary fiber intake, which shifts gut microbial metabolism toward amino acid fermentation from animal-derived foods. This process generates gut-derived uremic toxins, including p-cresyl sulfate, indoxyl sulfate, and trimethylamine N-oxide (TMAO) (254), which impair osteoblast differentiation, suppress bone formation, and reduce bone mineral density (255–257).

Dietary fiber represents a major nutritional deficiency in CKD patients. Studies in rat models of progressive CKD have confirmed that dietary inulin supplementation reshapes cecal microbiota alpha and beta diversity, increases the abundance of *Bifidobacterium* and *Bacteroides*, decreases the abundance of *Lactobacillus*, lowers circulating levels of gut-derived uremic toxins, serum phosphorus, and parathyroid hormone, alleviates aortic and cardiac calcification, left ventricular mass index, and myocardial fibrosis, and improves bone turnover and cortical bone parameters (258).

Plant-based diets and the Mediterranean diet can enhance gut microbial diversity, promote short-chain fatty acid (SCFA) production, mitigate inflammation, and reduce bone resorption (259, 260). In contrast, high-protein animal-based diets elevate the abundance of harmful taxa such as *Bacteroides* and *Alistipes*, reduce levels of beneficial bacteria including *Lactobacillus* and *Roseburia*, and exacerbate disturbances in bone metabolism (261).

Fecal microbiota transplantation (FMT), as a means of whole microbiota remodeling, provides a new idea of “ecological therapy” for metabolic bone diseases, but its application in this field is still in the early exploratory stage (262). Against the special pathological background of CKD, which is characterized by both systemic inflammation and toxin accumulation, interventional strategies need to be more prudent. The oral adsorbent AST 120 not only adsorbs uremic toxin precursors but also specifically reduces the abundance of toxin-producing microbiota (uncultured Erysipelotrichaceae, *Clostridium* sensu stricto), thus modulating the production of harmful metabolites at the source (263). In contrast, the effects of probiotics/prebiotics in CKD patients are complex and controversial: randomized controlled trials have shown that supplementation with specific probiotics in hemodialysis patients failed to reduce inflammatory markers and uremic toxin levels, and may even be accompanied by elevated serum indoxyl sulfate (264).

In conclusion, microbiota-targeted interventions regulate the gut-kidney-bone axis through multiple levels and mechanisms, providing a promising strategy for improving skeletal health. However, their therapeutic efficacy is highly influenced by strain specificity, host individual differences, and disease status. Although microbiota research is still in its infancy, we believe that as future research moves beyond the one-size-fits-all supplementation model and conducts large-scale, long-term randomized controlled trials, the management of metabolic bone diseases and CKD will enter a brand-new era of Microbiome Medicine.

## 9 Conclusions

Overall, intestinal dysbiosis contributes to the pathogenesis of chronic kidney disease-mineral and bone disorder (CKD-MBD) by disrupting the gut-kidney-bone axis. The underlying mechanisms include impaired intestinal barrier function, excessive accumulation of uremic toxins, imbalanced microbial metabolites, and disordered endocrine signaling. Gut microbiota profiles show both disease specificity and partial overlap across chronic kidney disease, rheumatoid arthritis, osteoarthritis, and osteoporosis, laying a foundation for microbiota-targeted intervention strategies in metabolic bone diseases and renal disorders.

However, the included literature has inherent limitations: most clinical studies are small-sample observational designs with high population heterogeneity and inconsistent microbiota detection methods; randomized controlled trials (RCTs) are scarce, and findings from animal studies cannot be fully extrapolated to humans. Critical research gaps remain in this field: there are no unified standards for strain selection, dosage, and duration of microbiota interventions; long-term safety data in CKD populations are insufficient. As a narrative review, this article has methodological constraints: no systematic review or meta-analysis was performed, and no quality assessment was conducted for the included literature.

Despite the above limitations, this review carries important guiding value. For clinical practice, the gut microbiota can serve as a non-invasive biomarker for CKD-MBD risk stratification, and dietary fiber supplementation, prebiotics, and probiotics represent potential adjuvant therapies to alleviate uremic toxin accumulation and improve bone metabolism. For future research, large-scale, long-term, multicenter RCTs are warranted to clarify causal relationships by focusing on strain-specific effects, personalized dietary interventions, and multi-omics integration. Translational studies linking animal models to clinical applications should be performed to accelerate the implementation of microbiota-targeted strategies in the clinical management of CKD-MBD.

Collectively, targeting the gut microbiota holds great potential to revolutionize the prevention and treatment system of CKD-MBD and related metabolic bone diseases.
